# Assessing the impact of nitrogen supplementation in oats across multiple growth locations and years with targeted phenotyping and high-resolution metabolite profiling approaches

**DOI:** 10.1016/j.foodchem.2021.129585

**Published:** 2021-09-01

**Authors:** J. William Allwood, Pilar Martinez-Martin, Yun Xu, Alexander Cowan, Simon Pont, Irene Griffiths, Julie Sungurtas, Sarah Clarke, Royston Goodacre, Athole Marshall, Derek Stewart, Catherine Howarth

**Affiliations:** a Environmental and Biochemical Sciences, James Hutton Institute, Invergowrie, Dundee DD2 5DA, Scotland, UK; b Institute of Biological, Environmental & Rural Sciences (IBERS), Aberystwyth University, Plas Gogerddan, Aberystwyth, Ceredigion SY23 3EE, UK; c Department of Biochemistry and Systems Biology, Institute of Systems, Molecular and Integrative Biology, University of Liverpool, Biosciences Building, Crown Street, Liverpool L69 7ZB, UK; d ADAS Gleadthorpe, Mansfield, Nottinghamshire NG20 9PD, UK; e School of Engineering and Physical Sciences, Institute of Mechanical, Process and Energy Engineering, Heriot-Watt University, Edinburgh EH14 4AS, Scotland, UK

**Keywords:** Metabolomics, Oats (*Avena sativa* L.), Nitrogen, Grain quality, β-glucan, Proteins and amino acids, Lipids, Avenanthramides

## Abstract

Oats (*Avena sativa* L.) are a healthy food, being high in dietary fibre (e.g. β-glucans), antioxidants, minerals, and vitamins. Understanding the effect of variety and crop management on nutritional quality is important. The response of four oat varieties to increased nitrogen levels was investigated across multiple locations and years with respect to yield, grain quality and metabolites (assessed via GC- and LC- MS). A novel high-resolution UHPLC-PDA-MS/MS method was developed, providing improved metabolite enrichment, resolution, and identification. The combined phenotyping approach revealed that, amino acid levels were increased by nitrogen supplementation, as were total protein and nitrogen containing lipid levels, whereas health-beneficial avenanthramides were decreased. Although nitrogen addition significantly increased grain yield and β-glucan content, supporting increasing the total nitrogen levels recommended within agricultural guidelines, oat varietal choice as well as negative impacts upon health beneficial secondary metabolites and the environmental burdens associated with nitrogen fertilisation, require further consideration.

## Highlights

• The response to nitrogen of 4 winter oat varieties in three field trials was analysed.
• A novel high-resolution method was developed to profile metabolite changes.
• Conditions that enhance yield do not necessarily result in higher nutritional value.
• Choice of variety is of equally high importance as the nitrogen levels applied.

## 1 Introduction

Oats, *Avena sativa* L., are a cereal crop cultivated largely across temperate regions of the world providing a source of food for human consumption and animal-feeds, as well as providing a key source of emollients to the cosmetics industry (Marshall et al., 2013). Oats are commonly consumed as whole grains and have gained increased attention in recent decades due to their nutritional balance and large number of health benefits (Martínez-Villaluenga & Peñas, 2017). They are renowned as a rich source of dietary fibre, in particular the soluble fibre β-glucan, as well as being high in antioxidants, minerals and vitamins. Compared with other cereals, oats are relatively high in protein and lipid and lower in carbohydrate (Welch, 2011).

Based on clinical studies, both the U.S. Food and Drug Administration and the European Food and Safety Agency have approved health claims for oat-derived foods regarding the ability of oat β-glucan to reduce blood cholesterol and the risk of cardiovascular disease (FDA, 1997, EFSA, 2010). Studies have also shown that oat rich diets can directly be correlated with reduced blood pressure, blood-sugar and insulin levels, and increased satiety thus reducing the prevalence of obesity, risk of cardiovascular disease and Type II diabetes (as reviewed by Martínez-Villaluenga & Peñas, 2017). Oat β-glucan modulates the gut microbiota, particularly those bacterial species that influence host bile acid metabolism and production of short chain fatty acids (Joyce, Kamil, Fleige, & Gahan, 2019). Other oat constituents that might have a direct or indirect health beneficial impact include phytosterols, phenolic compounds, tocols and saponins (Shewry et al., 2008). Oats are a rich source of antioxidants, such as ferulic and phytic acids, and a unique group of antioxidants known as the avenanthramides. Avenanthramides have been documented to reduce arterial inflammation and positively impact the regulation of blood pressure (Meydani, 2009). Consumption of oat products have also been associated with prevention of cancer and gastrointestinal disorders (Thies et al., 2014, Martínez-Villaluenga and Peñas, 2017). The major storage proteins of oats, avenins, are believed to be less immunogenic to sufferers of coeliac disease, making oats suitable for a gluten-free diet (Welch, 2011), although the processing environment must be clean of other cereals.

Nitrogen fertilisation is well recognised for enhancing crop yield, yet nitrogen is a considerable environmental burden in crop production, directly due to greenhouse gas emissions during fertilizer production, and indirectly due to inefficiencies in fertilizer uptake leading to eutrophication of aquatic ecosystems (Rütting, Aronsson, & Delin, 2018). Proper management of nitrogen inputs is critical to optimize both crop yield and quality. However, the effect of nitrogen application on oat development, grain yield and quality, are poorly understood compared to in wheat and barley (Finnan, Burke, & Spink, 2019). Nitrogen fertilisation impacts on a number of agronomic traits in oats including plant height, grain yield and milling quality (Givens et al., 2004, Browne et al., 2006, Ma et al., 2017). High levels of nitrogen application have been shown to increase lodging which can significantly reduce yield (Berry et al., 2004, Yan et al., 2017). Oats are thought to require significantly lower nitrogen input (Weightman, Heywood, Wade, & South, 2004), are considered to be effective sequestrators of nitrogenous compounds, perform well on land less suitable for wheat production, and have a lower ecological footprint compared to other arable crops (Sylvester-Bradley & Kindred, 2009). Oat varieties with greater nitrogen use efficiency or with a higher responsiveness to nitrogen (Swarbreck et al., 2019) that maintain high output, would reduce nitrogen input requirements and environmental impact, as well as increasing the crops economic desirability.

Despite its importance for the end-user and milling industry, oat milling quality and grain composition are not currently considered in UK agricultural guidelines for optimal nitrogen application levels. The selection of oat variety and crop management practices and their role in determining oat nutritional quality, are important areas which require further understanding. To meet these objectives, four winter oat varieties (Mascani, Tardis, Balado and Gerald) were grown in three replicated nitrogen response trials, over two locations and two years. The trials consisted of a no added nitrogen control and up to five added nitrogen treatments. Grain yield, yield components and grain quality traits were assessed, as were total lipid, protein and ß-glucan levels. The de-hulled oats (groats) were also subjected to Gas Chromatography – Mass Spectrometry (GC-MS) analysis of polar metabolites. A newly developed extraction enrichment and high resolution Ultra High-Performance Liquid Chromatography (UHPLC)-MS/MS method, superseding a previous rapid (sub 15 min) method (Allwood et al., 2019a), was applied to profile a range of lipids, phenolic compounds, and a number of primary metabolites retained by C18 UHPLC.

## 2 Material and methods

### 2.1 Field trial design

Three trials were conducted, the first at IBERS, Lydbury North, Shropshire, UK (latitude 52.45, longitude −2.94, sandy loam soil type) in 2014 (IBERS 2014), the second and third at ADAS Rosemaund, Herefordshire, UK (latitude 52.15, longitude −2.59, sandy clay loam soil type) in 2014 and 2015 (ADAS 2014 and ADAS 2015 respectively). The trials consisted of a split plot design with three replicates, using five (IBERS 2014) or six (ADAS 2014 and ADAS 2015) levels of nitrogen application (main plot treatment) and four commercially available winter oat varieties (sub-plot treatment). These included two of the most widely grown winter oat varieties in the UK over the last 20 years: Gerald and Mascani (AHDB, 2015). Three varieties were of conventional height (Mascani, Gerald and Tardis) and the fourth was a dwarf variety (Balado). Plots (1.8 × 6 m) were sown on the 9th October 2013 (IBERS 2014), 30th September 2013 (ADAS 2014) and 1st October 2014 (ADAS 2015), at a sowing rate of 300 seeds m^−2^ and harvested on the 7th August 2014 (IBERS 2014), 4th August 2014 (ADAS, 2014) and 8th August 2015 (ADAS 2015), using a small plot combine which incorporated a grain moisture analyser. Fungicides and weed control followed standard practise for winter oats. Soils were sampled to a depth of 90 cm in early spring and residual soil nitrogen was determined to be 96 kg N/ha (IBERS 2014), 22 and 24 kg N/ha (ADAS 2014 and ADAS 2015). Nitrogen (ammonium nitrate) doses were split between three different developmental stages in early to late spring to provide five-six final treatments (Table S1). Prior to harvest the number of fertile shoots (panicles) per m^2^ were counted. Lodging (% plot area at greater than 45° from vertical) and leaning (% plot area at 10° to 45° from vertical) were assessed immediately prior to harvest at all sites. These data were used to calculate a lodging index on a 0–100 scale: lodging index = % area lodged + (% area leaning)/2. A linear plus exponential model (George, 1984) was used to fit grain yield responses to N and the economically optimum N rate (Nopt) was determined assuming the breakeven price ratio to be 5:1 as per the England and Wales Fertiliser recommendations RB209, i.e. the crop yield (kg) needed to pay for 1 kg N.

### 2.2 Chemicals

Unless otherwise stated all solvents (methanol, water, acetonitrile and chloroform) were of HPLC grade and JT Baker brand (Scientific Chemical Supplies, UK), formic acid was of Ultima MS grade (Thermo Fisher Scientific, UK), dichloromethane, isohexane, pyridine, methoxyamine hydrochloride, *N*-Trimethylsilyl-*N*-methyl trifluoroacetamide (MSTFA), Sorbitol, Ribitol and *n*-alkanes (*n*-decane (C10), *n*-dodecane (C12), *n*-pentadecane (C15), *n*-octadecane (C18), *n*-nonadecane (C19), *n*-docosane (C22), *n*-octacosane (C28), *n*-dotriacontane (C32) and *n*-hexatriacontane (C36)), were obtained from Sigma-Aldrich UK.

### 2.3 Grain quality assays

Residual straw, unthreshed panicles, large debris and dirt, were cleaned from the grain using 3.5 and 2 mm sieves and the grain dried to 15% moisture content. The thousand grain weight (TGW) was determined using a seed counter (Data technologies model S-25). Grain numbers per m^2^ and per panicle were calculated using grain yield and TGW results. Groats were obtained by passing 25 g of whole grain through a Laboratory Oat Huller (Codema Model LH5095; Maple Grove, Minneapolis, USA) set at 100 bar for 60 s. Groat nitrogen and oil content were predicted using near infrared spectroscopy (NIRS) and β-glucan content was determined with a Megazyme™ kit, both as described in Allwood et al. (2019a). These data were analysed using 2-way analysis of variance and Fisher’s protected least significance test using GENSTAT 16th edition and correlation analysis conducted using Microsoft Excel.

### 2.4 Extraction of samples for UHPLC-PDA-MS

Groats were homogenised using a Retsch Cyclone Mill – Twister (12,000 rpm min^−1^; 62 milli second peripheral rotor speed; 1 mm sieve; 1 min cycle). A quality assurance (QA) sample was prepared from a mix of all individual samples. To 100 mg FW of groat flour 3 mL of extraction buffer (75% methanol 24.9% water 0.1% formic acid) was added, the sample was vortex mixed and ultrasonicated for 15 min, shaken for 30 min and centrifuged (3220 x*g*, 3 °C, 10 min). Two 1.2 mL aliquots of supernatant were dried to completeness by speed vacuum concentration and stored at −80 °C. Samples were reconstituted in 140 µL of HPLC grade methanol followed by 280 µL of HPLC grade water, prior to shaking for 30 min and centrifugation (3220 x*g*, 3 °C, 10 min). The supernatants were filtered (Thomson 0.45um PTFE filter vial) and transferred to HPLC vials with pre-slit caps (Thermo Fisher Scientific, Micro Sampling Vial 300 µL and 9 mm PTFE/SIL cap). The samples were transferred to the HPLC autosampler (10 °C) and each trials sample set analysed within 72 h.

### 2.5 UHPLC-PDA-MS and MS/MS analysis

A Thermo Dionex U3000 (HPG3200RS pump; TCC3000 column oven; WPS3000TFC autosampler) connected to a U3000 (VWD3000) PDA (Thermo Fisher Scientific UK), was applied to UHPLC separations. The flow rate was 300 µL/min, the column and guard column (Waters Cortecs C18 (RP18, ODS, Octadecyl) T3 1.6 µm particle 100x2.1 mm; Cortecs C18 T3 1.6 µm particle 5x2.1 mm VanGuard cartridge) were maintained at 40 °C, solvent A, HPLC grade water, and solvent B, HPLC grade acetonitrile, were acidified with 0.1% [v/v] formic acid, 10 µL of extract (1.9 mg on-column mass) was injected in part-loop mode. The gradient programme was as follows: 5–35% B 0–27 min, 35–80% B 27–32 min, hold 80% B 32–36 min, 80–5% B 36–36.1 min, hold 5% B 36.1–40 min. Acetonitrile:water 8:2 and methanol:water 1:9 were applied as the needle and rear-seal washes, respectively. The UHPLC eluent was monitored via the PDA in wavelength/absorbance mode 200–600 nm (1 nm filter bandwidth and wavelength step, 1 s filter rise time, 10 Hz sample rate).

The UHPLC eluent was transfered to a Thermo LTQ-Orbitrap XL MS operated under Xcalibur software (Thermo Fisher Scientific UK) from 0 to 36 min, prior to being diverted to waste from 36 to 40 min. Full-scan FT spectra (*m*/*z* 100–2000) were collected at 30,000 resolution (FWHM defined at *m*/*z* 400) in profile mode for all samples. Data-dependent analysis (DDA) was applied to obtain MS^2^ data with the QA sample (Allwood et al., 2019b), accross reduced scan ranges (100–400, 400–700, 700–1000 and 1000–2000 *m*/*z*) to maximise MS2 data capture. The DDA method applied a primary FT full scan, followed by a secondary LTQ-IT scan to collect MS^2^ on the top 3 most intense ions (CID, Helium collsion gas, 45% NCE, 30 ms activation time, 0.25 activation Q, singly charged ions only, isotopes excluded, 30 s dynamic exclussion). Tuning, calibration, scan speed, automatic gain control, and ESI source settings are available in Allwood et al. (2019b). For each analytical block, eight QA injections were performed for conditioning, followed by three QAs, eight experimental samples, and a further QA injection. This was repeated until all experimental samples were analysed and was concluded with a further three QA injections. Blank samples were analysed at the start and end and the analytical block concluded by collection of DDA MS2 of QA samples.

### 2.6 UHPLC-PDA-MS data processing, peak annotation and identification

The UHPLC-PDA-MS data were centroided and deconvolved with XCMS online (https://xcmsonline.scripps.edu/). XCMS generates an XY matrix containing paired RT and *m*/*z*, along with the extracted ion chromatogram (EIC) area, for each profiled sample. Peaks that eluted from 36 to 40 mins or that were dominant within blank sample extracts were excluded, as were isotopes and minor adducts. The neutral accurate mass is next calculated for each parent ion (<5ppm) and in turn matched to possible molecular formula(s) and metabolite name(s) applying PutMedID (Allwood et al., 2019b, Brown et al., 2011). Several metabolite libraries were applied, (i) metabolites reported in oats (https://foodb.ca/), (ii) plant metabolites (http://www.plantcyc.org), and (iii) the Manchester Metabolomics Database (MMD: http://dbkgroup.org/MMD/). To increase confidence in the assigned putative identification(s), QA sample MS/MS data were also matched to reference spectra (MassBank Europe (https://massbank.eu/MassBank/); FooDB (https://foodb.ca/); LipidMaps (https://www.lipidmaps.org/); de Bruijn et al., 2019, Wang et al., 2017, Liebisch et al., 2002, Suárez-García et al., 2017). Reference MS/MS spectral database identifiers and literature citations are provided in Supplementary Table S2 (ESI negative mode) and Table S3 (ESI positive mode).

### 2.7 GC-MS extraction and derivatisation

To 100 mg FW of groat flour (in glass tubes), 3 mL of methanol was added and mixed at 30 °C for 30 min. 100 µL of internal standard (2 mg/mL Sorbitol, 2 mg/mL Ribitol, in 1:1 methanol:water) and 0.75 mL of water were added and mixed at 30 °C for 30 min, before 6 mL of chloroform and further mixing at 30 °C for 30 min. 1.5 mL of water was added and the samples vortex mixed and centrifuged (3220 x*g*, 3 °C, 10 min). 0.5 mL of polar (upper) phase was dried via speed vacuum concentration. A retention index (RI) standard mixture of *n*-alkanes (*n*-decane (C10, RI 1000), *n*-dodecane (C12, RI 1200), *n*-pentadecane (C15, RI 1500), *n*-octadecane (C18, RI 1800), *n*-nonadecane (C19, RI 1900), *n*-docosane (C22, RI 2200), *n*-octacosane (C28, RI 2800), *n*-dotriacontane (C32, RI 3200) and *n*-hexatriacontane (C36, RI 3600)) was prepared at 0.23 mg/mL in pyridine. 40 µL of methoxyamine hydrochloride (40 mg/mL in pyridine) was added and the sample mixed at 30 °C for 1.5 h. 10 µL of retention index and 70 µL of MSTFA were added and the sample mixed at 37 °C for 30 min. The derivatised polar fraction was then transferred to autosampler vials with 300 µL fixed glass inserts and PTFE coated non-slit caps.

### 2.8 GC-MS analysis, data processing and peak identification

GC-MS analysis was performed with a Trace GC (PTV injector) and a DSQ II MS system operated under Xcalibur (Thermo Fisher Scientific, UK). 1 μL of derivatized sample was injected at 250 °C in split (1:30) mode with helium as the carrier gas (1.5 mL/min), post injection the purge flow was set to 20 mL/min for 1 min. The needle was washed before sample injection with dichloromethane followed by derivatized sample, and post-injection with dichloromethane followed by isohexane. Separations were performed with an Agilent DB-5MS capillary column (15 m, 0.25 mm i.d., 0.25 µm film, 10 m EZ-Guard), a 380 s solvent delay was applied, the GC program was initiated at 100 °C isothermal for 2.1 min, ramped to 320 °C at 25 °C/min, and held 3.5 mins at 320 °C isothermal, with the transfer line at 250 °C. The DSQ II MS system was operated in electron ionisation (EI) mode (70 eV) with a mass range of 35–900 *m*/*z* and scan rate of 6 scans/s, 200 °C source and 250 °C interface temperatures. Using Xcalibur, chromatographic deconvolution was performed (S/N threshold 10, baseline offset 1.0, data points for averaging 5, peak width 3). Metabolite peaks were identified by matching against an in-house mass spectral/RI library (MSI level 1: Sumner et al., 2007). Identifications (Table S4 GC-MS) were only considered as unambiguous if a +/- 10 RI match and MS match greater than 800 was attained compared to authentic reference compounds analysed under the same conditions and instrument.

### 2.9 Chemometric analysis of UHPLC-PDA-MS and GC-MS metabolite profiles

UHPLC-MS data were normalised to total ion current (TIC) and GC-MS data to the ribitol internal standard. The relative standard deviation (RSD) for each feature across the QAs was calculated, features with 25%> RSD were excluded from statistical tests. Principal Components Analysis (PCA) (SIMCA-P + 12.01: Umetrics, Umeå, Sweden) was applied to each of the seven UHPLC-MS and GC-MS datasets. In addition, multiblock (MB) hierarchical (H)PCA models (MB-HPCA; Smilde, Westerhuis, & de Jong, 2003) were generated as previously described (Biais, Allwood, Deborde, Xu, Maucourt, Beauvoit, Dunn, Jacob, Goodacre, Rolin, & Moing, 2009). The blocking design investigated the effect of nitrogen without regard to oat variety (variety blocking), and of oat variety without regard to nitrogen level (nitrogen blocking). The variable identifiers applied to the MB-HPCA loadings are given in Tables S2 (ESI negative), S3 (ESI positive), and S4 (GC-MS). Spearmans Rho, a non-parametric correlation coefficient (Kendall, 1970) was calculated using each experimental and technological dataset to select metabolites which highly correlated with the total nitrogen level. Clustergrams were generated based upon the methods of Eisen et al. (Eisen, Spellman, Brown, & Botstein, 1998) and within each clustergram hierarchical cluster analysis was applied to metabolic features, using Spearman Rho as the distance metric. A non-parametric two-way ANOVA, i.e. the Friedman test, was performed applying a False Discovery Rate (FDR) correction of 5% (Benjamini-Hochberg), to define metabolite changes based upon nitrogen level or oat variety. All statistical analyses were performed in MATLAB R2018a, the Friedman test and Spermans Rho calculation applied the statistics and machine learning toolbox (Mathworks, MA, US), MB-HPCA applied freely available in-house scripts (https://github.com/Biospec). Heat maps illustrating oat varietal differences were generated based upon log10 scaled normalised peak areas of significant metabolite features with MultiExperimentViewer (http://mev.tm4.org/).

## 3 Results and discussion

### 3.1 Oat yield and grain quality

**Fig. 1:**
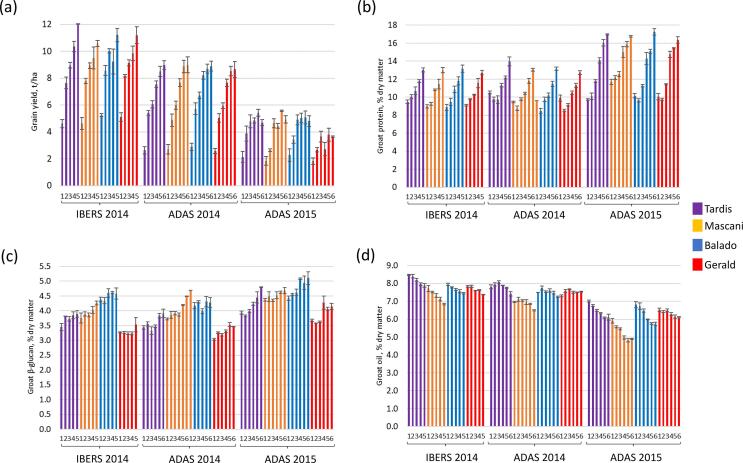
Response of mean (a) grain yield (t/ha at 15% moisture content) +/- standard error), (b) groat protein (% dry matter), (c) groat β-glucan (% dry matter) and (d) groat oil (% dry matter), to nitrogen application for four winter oat varieties at three field trials, IBERS 2014, ADAS 2014 and ADAS 2015. Nitrogen level 1: 0 kg N/ha applied; level 2: 50 kg N/ha applied (2014 trials) and 60 kg N/ha applied (2015 trial); level 3 100 kg N/ha applied (2014 trials) and 120 kg N/ha applied (2015 trial); level 4 150 kg N/ha applied (2014 trials) and 180 kg N/ha applied (2015 trial); level 5 200 kg N/ha applied (2014 trials) and 230 kg N/ha applied (2015 trial); level 6 250 kg N/ha applied ADAS 2014 and 280 kg N/ha applied ADAS 2015). Replication *n*3.

The application of nitrogen significantly enhanced grain yield across all three trials (Fig. 1a), but there was only a significant difference between the four varieties at the ADAS 2015 trial (Table 1). Although grain yields did not continue to increase significantly beyond the addition of 200 kg N/ha, optimal levels could only be calculated for ADAS 2015 because for the other two trials the yields were continuing to increase above the highest level of nitrogen applied. The calculated economic optimum N rate was highest for Mascani at 207 kg N/ha and lowest for Gerald at 160 kg N/ha. The response to nitrogen for grain yield of Balado and Tardis were very similar with an economic optimum of 170 kg N/ha.

**Table 1:** Mean values by variety and by nitrogen application level for grain yield (t/ha at 15% moisture content), lodging Index and yield component traits (panicle number per m^2^, grain number per m^2^ and grain number per panicle). Replication *n*3. Different letters indicate significant differences between mean values at p < 0.05 as calculated by Fisher’s least significance difference test. Nitrogen treatment and Variety *p* values (and their interaction) and the level of significance of these factors as calculated by two-way ANOVA. IB14: IBERS 2014 trial; AD14: ADAS 2014 trial; AD15: ADAS 2015 trial.

	Grain yield (t/ha)@ 85 % Dry Matter			Lodging Index			Thousand Grain Weight (g)			Grain n^o^/m^2^			Panicles/m^2^			Grain n^o^ per panicle		
	IB14	AD14	AD15	IB14	AD14	AD15	IB14	AD14	AD15	IB14	AD14	AD15	IB14	AD14	AD15	IB14	AD14	AD15
**Variety**																		
Balado	8.86	6.86	4.25b	0.00b	0.00b	0.00	43.76c	43.71c	40.33c	17129b	13301b	9006ab	300.2a	300.1a	400.9a	56.64d	43.40d	22.05c
Gerald	8.70	6.26	3.06a	9.40a	8.33a	0.00	38.94a	36.12a	32.69a	18943c	14867d	8014ab	392.0bc	378.5b	432.7a	48.28c	38.22c	18.38ab
Mascani	8.29	6.52	4.01b	0.00b	3.89ab	0.00	45.85d	45.52d	42.49d	15308a	12117a	7938ab	399.8c	415.2b	502.7b	37.98a	28.55a	15.45a
Tardis	8.72	6.52	4.26b	10.92a	2.22b	0.00	42.57b	41.41b	38.30b	17564b	13310c	9628b	363.2b	406.3b	482.5b	47.82b	32.14b	19.57bc
**Nitrogen level**																		
1	4.90a	2.70a	2.02a	0.00a	0.00a	0.00	41.49a	41.10a	38.29ab	10095a	5580a	4487a	251.8a	278.6a	321.9a	40.40a	20.52a	13.92a
2	8.05b	5.26b	2.99b	0.00a	0.00a	0.00	44.11c	41.92ab	39.37b	15603b	10724bc	6844b	353.7b	358.5b	425.6b	44.82b	31.17b	16.27ab
3	9.26c	6.17c	4.50c	1.04a	0.00a	0.00	42.96bc	41.38ab	39.45b	18427c	12773ab	9732c	414.6c	378.3bc	482.1c	45.98bc	34.70bc	20.57bc
4	9.74c	7.77d	4.27c	6.00a	1.67a	0.00	42.03ab	42.01ab	38.27ab	19772d	15860bc	9401c	408.1c	422.6c	480.0c	49.17c	38.30c	19.92bc
5	11.25d	8.65e	4.97c	18.54b	3.75a	0.00	43.32c	42.84b	38.08ab	22280e	17302c	11084c	390.8c	404.1bc	514.0c	58.03d	43.65d	21.74c
6		8.90e	4.52c		16.25b	0.00		41.30a	37.27a		18453ab	10335c		407.9bc	504.6c		45.76d	20.75c
N treatment p value	<0.001	<0.001	<0.001	<0.001	<0.001		<0.001	0.001	0.001	<0.001	<0.001	<0.001	<0.001	<0.001	<0.001	<0.001	<0.001	<0.001
Variety (V) p value	0.164	0.132	<0.001	<0.001	<0.001		<0.001	<0.001	<0.001	<0.001	<0.001	0.002	<0.001	<0.001	<0.001	<0.001	<0.001	<0.001
V x N p value	0.290	0.990	0.191	<0.001	<0.001		0.015	0.094	<0.001	0.101	0.024	0.826	0.506	0.451	0.079	0.114	0.147	0.815

The grain yield of a cereal crop like oats can be split into three major components: the panicle population density, the number of grains per panicle and the individual grain weight. In all three trials, although thousand grain weight (TGW) was significantly affected by nitrogen (Table 1) it was not correlated with grain yield. Grain yield was however highly correlated with the number of grains m^−2^ (*r* = 0.99). The grain number m^−2^ is a combination of panicle number m^−2^ and grain number per panicle, both of which increased significantly as nitrogen increased in all trials (Table 1). These results suggest the productive tiller survival rate increases as greater levels of nitrogen are provided, as previously reported (Browne et al., 2006, Ma et al., 2017, Weightman et al., 2004, Finnan et al., 2019). However, it was found in this study that the panicle number m^−2^ did not increase beyond applications of 100 kg N/ha, and that the grain number per panicle continued to increase with higher applications of nitrogen (Table 1). Oats display phenotypic plasticity in response to soil-climate conditions, but this is strongly influenced by variety. Although differences in total grain yield between oat varieties were only significant at ADAS 2015, significant differences were found for the three yield component traits (Table 1). There was however no significant interaction between variety and nitrogen treatment for these traits. Mascani was observed to have the lowest grain number per panicle and the highest panicle number m^−2^ as well as the highest TGW under all treatments, whereas Balado had the highest grain number per panicle and lowest panicle number m^−2^ in all three trials. Large increases in grain numbers with nitrogen fertilisation have been found previously (Peltonen-Sainio and Peltonen, 1995, Finnan et al., 2019). This may either be due to increased initiation or survival of spikelet primordia. When a greater number of grains are formed, competition can result in incomplete grain filling, reducing the final grain size (Marshall et al., 2013). The results suggest that whilst the different oat varieties can change yield component structure in response to changing nitrogen levels, the actual grain yield remains unchanged (Fig. 1).

Increased grain yield at high nitrogen application rates has previously been found to result in crop lodging which can reduce grain quality (Berry et al., 2004, Yan et al., 2017). No lodging was observed in the ADAS 2015 trial but a significant effect of nitrogen and of variety was found in both 2014 trials (Table 1). There was a highly significant interaction between variety and nitrogen treatment with the dwarf variety, Balado, displaying little lodging even at high levels of nitrogen application.

Although TGW did not correlate with grain yield in any of the trials and there was not a linear relationship between increased nitrogen level and TGW, it was significantly different between varieties and nitrogen levels (Table 1). A significant interaction between variety and nitrogen treatment was also found. Mascani not only had the highest mean TGW in each trial, but its TGW also increased with the addition of nitrogen, whereas Gerald had the lowest TGW across each trial and its TGW decreased with increasing levels of nitrogen. Balado and Tardis displayed a varying response depending on the trial, with their TGW decreasing in ADAS 2015 at higher nitrogen levels, and a less clear effect revealed in the other two trials (Table 1). Previous studies have shown that increasing nitrogen application results in lower individual grain weight (Ma et al., 2017, Peltonen-Sainio and Peltonen, 1995, Weightman et al., 2004) but this study suggests that the response of TGW to nitrogen is oat variety dependent. TGW is often used as an indicator of grain quality since it is related to grain plumpness, with high values reflecting well-filled grains.

### 3.2 Improvement of oat grain metabolite separation, annotation and identification, via extract enrichment and high resolution UHPLC-PDA-MS/MS

**Fig. 2:**
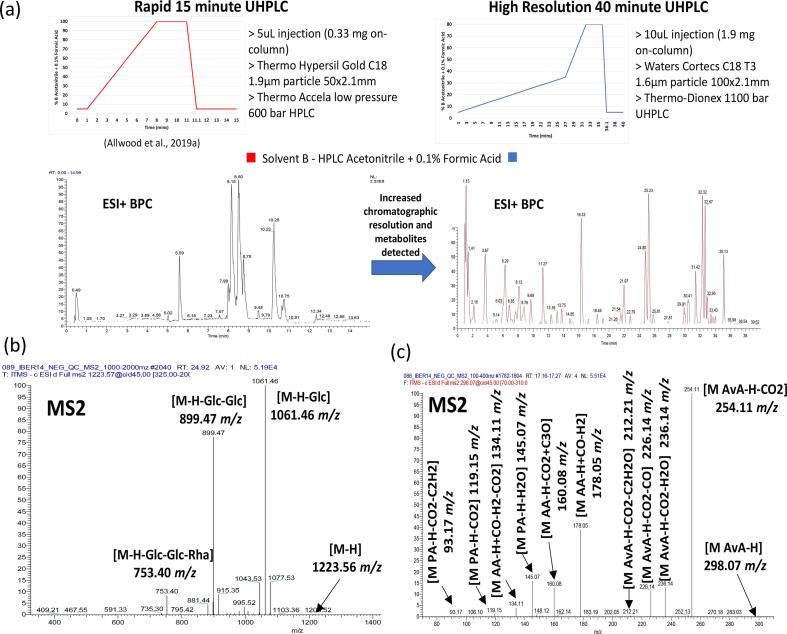
Improvements in oat grain UHPLC-MS methodology (a) and example MS/MS identifications of Avenacoside B (b) and Avenanthramide 2p (A) (c). MS/MS Avenacoside B and Avenanthramide 2p (A) annotations based upon Wang et al., 2017, de Bruijn et al., 2019, respectively. BPC Base Peak Chromatogram; Glc Glucose; Rha Rhamnose; AvA Avenanthramide A; AA Anthranilic acid; PA Phenylalkenoic acid.

The UHPLC-PDA-MS metabolite profiling method generated extremely rich metabolite profiles, representing a wide-array of classes, including primary metabolites such as amino acids, TCA intermediates, sugars and lipids, as well as secondary metabolites including saponins such as the avenacins and desglucoavenacosides, and phenolic compounds such as the avenanthramides and avenanthramide-hexosides (Table S2 and S3). In addition, GC-MS provided a complementary approach better tailored to the retention of a wider range of amino acids and sugars (Table S4). Several updates to our previous oat UHPLC-PDA-MS method (Allwood et al., 2019a) have been made, including: 5.75-fold increase from 0.33 mg to 1.9 mg of groat material on-column; higher resolution column and high pressure capacity UHPLC system; and application of a longer gradient. As can be seen from the representative UHPLC chromatograms (Fig. 2a), these updates have resulted in the detection and resolution of many more metabolites.

The deconvolution of the UHPLC-PDA-MS profiles within XCMS online generates highly information rich datasets (putative molecular features (RT-*m*/*z* pairs): IBERS 2014 ESI + 14,915, ESI- 20,646; ADAS 2014 ESI + 16,117, ESI- 19,297; ADAS 2015 ESI + 18,847, ESI- 22,008). After filtering (removal of blank extract, void and equilibration peaks), Pearson correlations were applied to group *m*/*z* features likely to be associated with the same compound (i.e. a RT-*m*/*z* group). Within each RT-*m*/*z* group, accurate mass differences were calculated between peaks, and in-turn the peaks were annotated as parent, isotope and adduct ions (e.g. ESI + [M+H], [M+Na], [M+K], [M+NH3], [M+HCOOH]; ESI- [M-H], [M+HCOOH-2H], [M+Cl]), as well as common in-source fragments. The neutral accurate mass was calculated and matched to a library of molecular formulae and metabolite names. Experimental MS/MS spectra were also matched to those available in the literature for avenacosides (Wang et al., 2017) (Fig. 2b), avenanthramides and avenanthramide-hexosides (de Bruijn et al., 2019) (Fig. 2c), and lipids (Liebisch et al., 2002, Suárez-García et al., 2017) (Tables S2 and S3). The RT-*m*/*z* groups were further filtered to remove redundant isotope, adduct and in-source fragment features, preserving only the most intense RT-*m*/*z* for each metabolite or unknown feature. The finalised filtered and QA’d ESI negative and positive mode datasets contained 439 and 481 features, respectively. The GC-MS data was far less complex with a total of 62 metabolite features, including 10 unknown (MSI level 4) and 52 identified metabolites (MSI level 1), 59 of which passed QA (Table S4).

### 3.3 Chemometric analysis of UHPLC-PDA-MS and GC-MS oat grain metabolite profiles

The UHPLC-PDA-MS and GC-MS datasets were first assessed applying standard PCA models (Figure S1). The samples revealed a distribution across the PC1 axis associated with increased nitrogen application, other than for the ADAS 2015 trial ESI negative mode dataset, where samples revealed a distribution across PC2. The PC score plots indicated that Tardis, Mascani and Gerald possessed closely related metabolic phenotypes, whereas Balado, the dwarf oat variety, was far more distinct.

To consider metabolite trends associated individually with oat variety or nitrogen application level, MB-HPCA models were generated for each trial with two blocking statements applied. The first blocking statement separated the oat varieties into individual blocks and revealed the common trends associated with increasing nitrogen levels (Figure S2a, S3a, S4a), whereas the second blocking statement separated the various nitrogen levels into individual blocks and revealed the oat varietal differences (Figure S2c, S3c, S4c). Individual MB-PCA models were generated for each field trial and technological dataset, within each model, the common trends from each individual PCA block scores plot are combined into a ‘super scores’ plot and the significant metabolites associated with the trends selected through a block weighted averaged ‘block loadings’. Based upon the oat variety blocking statement, the super scores and loadings plots were generated for each trial, IBERS 2014 (Figure S2b), ADAS 2014 (Figure S3b) and 2015 (Figure S4b). The PC loadings significance cut-off for metabolites that were increased in concentration in response to increased nitrogen are indicated by a red arrow, and metabolites that were decreased in concentration are indicated by a blue. Based upon the nitrogen level blocking statement, the super scores and loadings plots were generated for each trial, IBERS 2014 (Figure S2d), ADAS 2014 (Figure S3d) and 2015 (Figure S4d), the PC loadings significance cut-off for metabolites that differentiated the Balado variety are indicated by a blue arrow, and metabolites that differentiated the non-dwarf varieties are indicated by a red arrow.

**Fig. 3:**
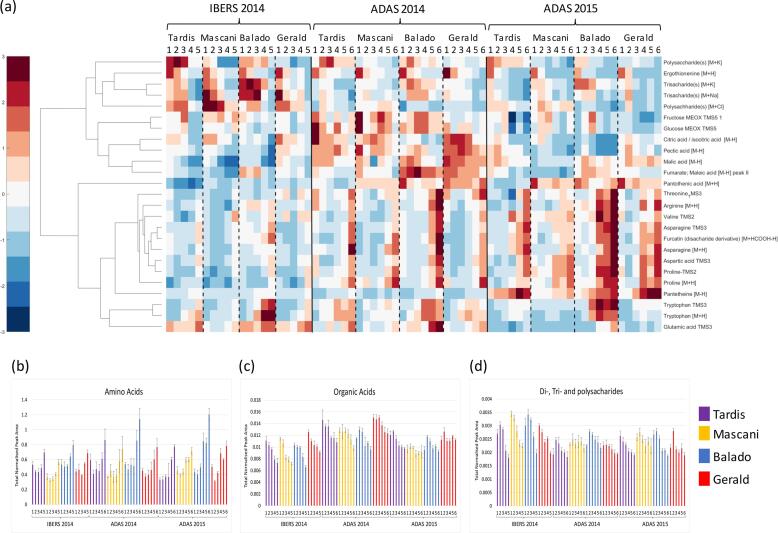
Central metabolite responses to increasing nitrogen level: (a) hierarchical cluster analysis applied to the response of metabolic features to nitrogen using Spearman Rho as the distance metric. Normalised values were scaled (+3 > -3) and a false colour code applied to generate the heat map; (b) Bar plot of total amino acids responding to nitrogen; (c) Bar plot of total organic acids responding to nitrogen; (d) Bar plot of di-, tri- and poly-saccharides responding to nitrogen. Error bars represent the standard error. Replication *n*3.

**Fig. 4:**
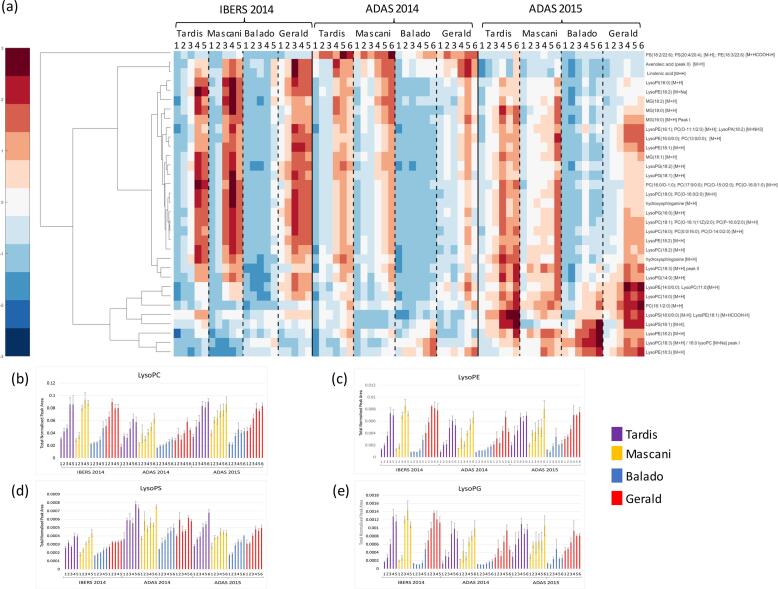
Lipid responses to increasing nitrogen level: (a) hierarchical cluster analysis applied to the response of metabolic features to nitrogen using Spearman Rho as the distance metric. Normalised values were scaled (+3 > -3) and a false colour code applied to generate the heat map; (b) Bar plot of total Lyso PC’s responding to nitrogen; (c) Bar plot of total Lyso PE’s responding to nitrogen; (d) Bar plot of total Lyso PS’s responding to nitrogen; (e) Bar plot of total Lyso PG’s responding to nitrogen. Error bars represent the standard error. Replication *n*3.

**Fig. 5:**
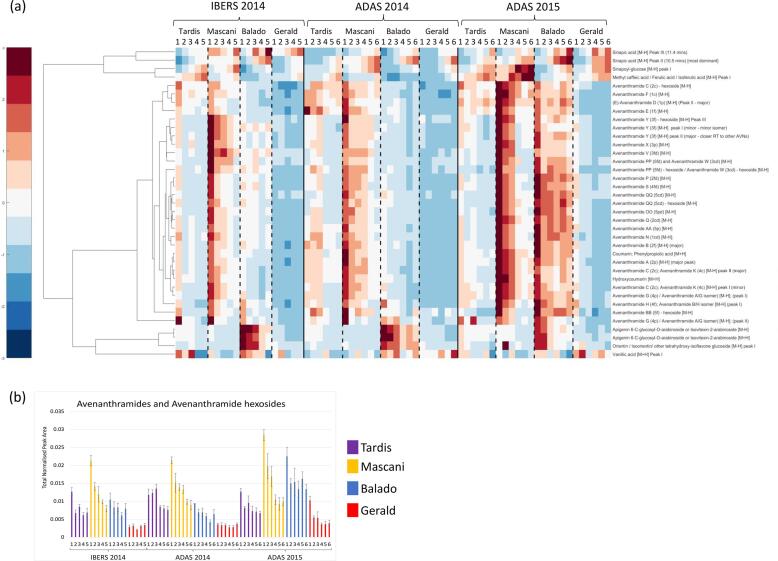
Secondary metabolite responses to increasing nitrogen level: (a) hierarchical cluster analysis applied to the response of metabolic features to nitrogen using Spearman Rho as the distance metric. Normalised values were scaled (+3 > -3) and a false colour code applied to generate the heat map; (b) Bar plot of total avenanthramides and avenanthramide-hexosides responding to nitrogen. Error bars represent the standard error. Replication *n*3.

With respect to metabolites that responded to nitrogen, Spearmans Rho coefficients were calculated using each trial and technological dataset to indicate which metabolites were most highly correlated, positively or negatively, with nitrogen level. Clustergrams were generated to show the most significantly correlated metabolites with respect to metabolite class, central metabolites (Fig. 3), lipids (Fig. 4) and secondary metabolites (Fig. 5).

Finally, a non-parametric univariate significance, the Friedman test (5% FDR), was performed. The Friedman test indicated: (i) metabolites that were significant with respect to nitrogen level, and (ii) metabolites that were significant with respect to oat variety (Table S2 UHPLC-MS negative mode, S3 UHPLC-MS positive mode, S4 GC-MS). Metabolites that were selected via MB-HPCA and which revealed univariate significance (*p* 0.01), as well as being significant for at least two of the three nitrogen trials, were further investigated. With respect to response to nitrogen, those metabolites that showed the highest Spearmans Rho coefficients, as well as greatest impact upon the MB-HPCA loadings (variety blocking) and high levels of univariate significance, were given the most focus.

### 3.4 Impacts of nitrogen supplementation on the primary metabolism of oat grains

Total protein levels significantly increased (*p* < 0.001) with the addition of nitrogen (Fig. 1b) and, unlike grain yield, continued to increase in a linear manner up to the highest nitrogen application levels. In the ADAS 2014 and 2015 trials there was also a significant difference between the four oat varieties (*p* < 0.001). The highest values were obtained at the lowest yielding trial, ADAS 2015, with a maximum value of 17.25% protein for Balado at the 280 kg N/ha treatment. In the ADAS 2015 trial there was a significant interaction between nitrogen and variety, with Mascani having higher protein compared to the other varieties at the lowest nitrogen levels. The β-glucan content was significantly different between varieties and also significantly increased (*p* < 0.001) with nitrogen application in all three trials (Fig. 1c), with Balado displaying the highest average value and Gerald the lowest. There was however no significant increase in β-glucan content at the higher N application rates. Previous studies reporting the effects of nitrogen application on β-glucan content have given conflicting results (Brunner and Freed, 1994, May et al., 2004, Redaelli et al., 2015, Saastamoinen et al., 1992, Yan et al., 2017). This is partly due to the different levels of nitrogen used in these various studies and also suggests that both genotype and environmental conditions are potentially having a great influence on this trait.

Corresponding with this increase in total protein levels, the first major impact upon oat grain metabolism was the up-regulation of amino acids. Asparagine, proline, tryptophan, valine, arginine, threonine, glutamic acid, hydroxy-tryptophan and aspartic acid, all increased as nitrogen addition increased (Fig. 3). Apart from glutamic acid, all of the aforementioned amino acids were also present at higher levels within the dwarf oat variety, Balado. Previous studies on the leaf tissues of wheat applying GC-MS analysis of MOX-TMS derivatives, revealed that a very similar range of amino acids were elevated when wheat plants were grown in the presence of nitrate in contrast to growth in its absence (Allwood et al., 2015). Nitrogen assimilation and amino acid metabolism are dependent upon a supply of NAD(P)H and/or reduced ferredoxin to serve as a reductant, as well as elevated levels of organic acids and ATP to supply a source of carbon skeletons and energy, respectively (Stitt et al., 2002). Although ATP and NAD(P)H can be directly assimilated via photosynthesis, mitochondrial respiration can also provide a source under certain conditions (Nunes-Nesi, Fernie, & Stitt, 2010). Carbohydrates are converted into organic acids via respiration (glycolysis, the TCA cycle and the oxidative pentose phosphate pathway), thus providing an alternative source of carbon skeletons. However, this results in nitrate assimilation competing directly with the Calvin cycle for reductant and ATP (Stitt et al., 2002). Within this study several TCA intermediates, namely citrate/isocitrate, aconitic acid, fumarate and malate (Fig. 3), were seen to decrease under conditions of high levels of nitrogen supplementation. These results suggest that TCA intermediates are being deplenished to increase the levels of ATP and reductant available for nitrogen assimilation. The depletion of a number of di-, tri- and polysaccharides was also observed as nitrogen application increased (Fig. 3), potentially highlighting their breakdown during mitochondrial respiration to elevate the availability of carbon skeletons for amino acid metabolism. These results indicate the intrinsic links and need for co-ordination between the carbon and nitrogen assimilation pathways to prevent severe imbalances. To investigate this further, measurements within the developing green tissues as well as the developing oat grain are required, to confirm which tissues act as the source and sites of import.

Several further interesting impacts upon primary metabolism were observed within this study. Intriguingly, the amino acid, ergothioneine, was observed to act conversely to all other measured amino acids, showing an extremely linear reduction in concentration as the level of nitrogen uptake was increased (Fig. 3). Ergothioneine is anabolised by a very limited range of bacterial and fungal species, it is therefore tempting to hypothesise that increased nitrogen levels are negatively impacting the rhizosphere population, or alternatively that due to the high availability of nitrogen, the oat plant has lower demands to assimilate ergothioneine. The imidazole, allantoin, was revealed to show an extremely linear increase as nitrogen supplementation increased, given that allantoin contains four atoms of nitrogen and has a key role in nitrogen cycling, this may simply reflect the greater availability of nitrogen for its metabolism, or possibly that the increased addition of nitrogen is resulting in oxidative stress and the potential degradation of purines. Pantetheine (vitamin B_5_) was also observed to increase in concentration in response to nitrogen, concurrently with a reduction in pantothenic acid levels (Fig. 3).

### 3.5 Nitrogen supplementation in oat grains up-regulates nitrogen containing membrane lipids but reduces total oil content

The total oil content was significantly different between varieties (*p* < 0.001) and decreased significantly in response to nitrogen (*p* < 0.001) across all three trials (Fig. 1d). A similar response has been found in spring oats (Yan et al., 2017). Mascani was revealed to have the lowest total oil content of the four oat varieties across all three trials and Tardis had the highest. There was a significant interaction of variety and treatment at both the ADAS 2014 (*p* = 0.009) and ADAS 2015 (*p* = 0.002) trials with the oil content of Gerald being relatively stable across nitrogen treatments. Total oil content was negatively correlated with both total protein content and β-glucan content. Conversely, increases in the levels of a range of nitrogen containing phospholipids as nitrogen levels increased were observed, especially lyso-phospholipids (Fig. 4). Large numbers of both lyso-phosphatidylcholines (PCs) (Fig. 4a and b) and lyso-phosphatidylethanolamines (PEs) (Fig. 4a and c), as well as a lesser number of lyso-phosphatidylserines (PSs) (Fig. 4a and d), were observed to increase under high levels of nitrogen supplementation. The lyso-phospholipids possessed 14:0, 16:0, 18:0, 18:1, 18:2 and 18:3 fatty acids, widely known as being the most abundant fatty acid species within the cellular membranes of plants. The fact that the majority of the lyso-phospholipids that increased in concentration possess nitrogen containing headgroups, is unlikely to be merely coincidental. Interestingly, the non-nitrogen containing lyso-phosphatidylglycerols (PGs) (Fig. 4a and e) were also observed to increase in concentration in response to nitrogen. The free fatty acids, linolenic acid (18:3) and avenoleic acid (hydroxy-linoleic acid), as well as a number of monoacylglycerols (MGs) with 16:0, 18:0, 18:1 and 18:2 fatty acyl constituents, were also increased in response to nitrogen (Fig. 4a). Hydroxy-sphingosine and hydroxy-sphinganine (Fig. 4a), 18-carbon amino alcohols and core components of cellular membrane sphingolipids, were also observed to increase in concentration in response to nitrogen. This range of observations underlines that very high levels of 18-carbon chain length lipid modulation are occurring in response to nitrogen elevation.

Given these results, it would be easy to assume that the oat grain total oil content would also increase under elevated nitrogen conditions. However, the total oil content was slightly decreased (Fig. 1d). Phospholipids, sphingolipids and MGs, do not alone represent the majority of lipid species within oat grains and it is perhaps the case that whilst nitrogen supplementation increases the synthesis of nitrogen containing phospholipids and sphingolipids, that the demands that their synthesis makes on alternative lipid species such as di- and tri- acyl glycerols (DGs and TGs) to provide fatty acid constituents, actually results in the slight overall decrease of total oil content. It is also likely that the extraction of oat grains in methanol rather than a non-polar solvent, combined with a UHPLC gradient that was finalised in 80% acetonitrile, limited the detection of the more non-polar oat grain lipid components.

### 3.6 Nitrogen supplementation has negative impacts on the levels of health beneficial avenanthramides

Increasing the supply of nitrogen was also seen to impact upon areas of secondary metabolism, especially with regards to a range of oat grain phenolic compounds and saponins. Compounds that increased in concentration in response to nitrogen, included, sinapic acid, sinapoyl-glucose, methyl-caffeic acid/ferulic acid/isoferulic acid, desglucoavenacoside A, and quinoline/isoquinoline (Fig. 5a). Given the antioxidant, antimicrobial, anticancer and antidiabetic activities reported for caffeic acid and its methyl and ethyl esters, as well as sinapic acid and its sinapoyl-glucose derivatives, these observations could be regarded as important with respect to oat grain derived health benefits. Conversely, several health beneficial phenolic compounds, namely the avenanthramides and their hexosides (Fig. 5a and b), were seen to be reduced in concentration as nitrogen supplementation increased. The oat grain avenanthramides, 1p (D), 2p (A), 1f (E), 2f (B), 3f (Y), 1c (F), 2c (C), 5p (AA), 1 cd (N), 2 cd (Q), 5 cd (QQ), 3p (X), 5pd (OO), 2fd (P), 4fd (S) (identified by accurate mass and MS/MS fragmentation; de Bruijn et al., 2019), as well as 3fd (V), 2c (C)/4c (K), and a series of minor 2p (A)/4p (G), 2f (B)/4f (H), 2pd (O)/4pd (R) isomers (identified by accurate mass alone), were all observed to decrease in concentration in response to nitrogen (Fig. 5a). In addition, a series of hexosides of avenanthramide 2c (C), 3f (Y) (identified by accurate mass and MS/MS fragmentation; de Bruijn et al., 2019), 5f (BB), 5fd (PP), 5 cd (QQ) and 3f (Y) (identified by accurate mass alone), were also observed to decrease in concentration in response to nitrogen (Fig. 5a). Reduced levels of avenanthramides and their hexosides potentially result from the high levels of up-regulation observed in primary metabolism, which effectively limits precursor availability for the metabolism of avenanthramides within both the oats green tissues and the grain. Interestingly, Gerald showed the lowest levels of the avenanthramides and a lesser reduction in their response to nitrogen, whereas Mascani, Tardis and Balado, showed the highest avenanthramide levels, and a much greater reduction in response to nitrogen (Fig. 5a and b).

### 3.7 Discrimination of oat variety based upon GC-MS and UHPLC-PDA-MS metabolite profiles

Although the primary aim of the field trials was to study the effect of increased nitrogen supplementation, a secondary aim of considering oat varietal differences was also investigated. By applying MB-HPCA with a blocking statement based upon nitrogen level, it was possible to identify oat varietal differences in metabolism. Within the MB-HPCA scores plots (Figure S2c, S3c and S4c), Balado, the dwarf oat variety, is clearly differentiated from Mascani, Tardis and Gerald. Mascani and Tardis are closely related in metabolic phenotype, showing near clustering, whilst Gerald also shows clear differences in metabolic phenotype. This reflects the pedigree relationships between these varieties with Mascani and Tardis being more closely related genetically than the other varieties (Tinker & Deyl, 2005; https://triticeaetoolbox.org/POOL/). When the MB-HPCA loadings that differentiate Balado (Figure S2d, S3d and S4d, loadings cut off indicated with a blue arrow) were investigated, higher levels of, amino acids (including valine/isovaline, tryptophan, hydroxy-tryptophan and asparagine), organic acids (succinic and fumaric acid), phenolic compounds and saponins (caffeic acid, O-feruloyl quinate, sinapoyl-glucose, vitexin/isovitexin (and their arabinosides), orientin/isoorientin (and their arabinosides), apigenin, avenacoside B2, vicenin 2 / vitexin-glucoside, and vulgaxanthin 2) (Figure S5), were observed in Balado. When the MB-HPCA loadings that differentiate Mascani, Tardis and Gerald (Figure S2d, S3d and S4d, loadings cut off indicated with a red arrow) were investigated, higher levels of, MGs, Lyso PCs, Lyso PEs, Lyso PIs, Lyso PGs, hydroxy sphingosine and hydroxy sphinganine, were observed in all three non-dwarf varieties (Figure S5). Tardis and Mascani, were also found to be higher in the levels of a number of saponins and phenolic compounds, including, methyl-caffeic acid/ferulic acid/isoferulic acid, avenacin A1 and 26-desglucoavenacoside A (Figure S5). With respect to the suite of avenanthramides and their hexosides, Mascani revealed the highest levels, followed by Tardis, with Gerald possessing the lowest levels (Figure S5).

## 4 Conclusions

The three nitrogen response field trials conducted at Lydbury North (IBERS 2014) and ADAS Rosemaund (ADAS 2014 and 2015), have revealed robust changes associated with winter oat grain yield and quality, metabolite levels, total protein, lipid and ß-glucan levels, with respect to increased levels of nitrogen supplementation and oat variety. They also reveal that conditions that result in higher yield do not necessarily result in higher grain quality or nutritional value. Although nitrogen addition significantly increased grain yield and β-glucan content, some traits, such as β-glucan, total oil content and a number of metabolites were affected by the variety used as much as by the addition of nitrogen. There were also significant variety by treatment interactions for a number of traits indicating that variety specific responses to nitrogen were obtained. This emphasises the importance of variety choice as well as management regime.

The recently developed metabolite extract enrichment and UHPLC-PDA-MS/MS profiling methods have achieved greater levels of metabolite chromatographic resolution, as well as greater numbers of metabolites detected and reliably identified, in comparison to our previous rapid UHPLC-MS method (Allwood et al., 2019a). Both the GC-MS and UHPLC-MS profiling approaches, through applications of high levels of QA, have been shown to be highly repeatable and appropriate for the screening of large numbers of oat grain samples. A number of interesting insights have been gained from the study and validated across multiple field trials, from changes in primary metabolism such as those observed to impact upon amino and organic acids, as well as the TCA cycle, through to the remodulation of lipid metabolism being directed towards the increased production of nitrogen containing membrane lipids. With respect to phenolic compounds, both positive (increased levels of caffeic and sinapic acids), and negative impacts (reduced levels of avenanthramides and their hexosides), that have influence upon the nutritional value of oats, were observed. This study has revealed that in addition to the significant enhancement of grain yield observed with increased nitrogen application, that there are a number of metabolic consequences, that vary between oat variety, for a wide range of metabolites that impact on the nutritional quality of the grain.

Although nitrogen addition significantly increased grain yield and β-glucan content, thus supporting increasing the total nitrogen levels recommended within current agricultural guidelines, especially with respect to the potential to increase profits for the farmer, the negative impacts upon health beneficial secondary metabolites and the environmental burdens associated with nitrogen fertiliser production and run-off, still require further consideration. The variability in phenotypic and metabolite profiling results between the different oat varieties, highlights that the choice of variety is of equally high importance as the nitrogen levels applied in the management regime. Future experiments under controlled conditions, sampling green tissues, as well as oat grains, throughout the development of the plant and grain, would reveal greater insight of carbon and nitrogen metabolism balance, and enable tracking of resource partitioning into lipid and secondary metabolism. Controlled studies will be key to our mechanistic understanding of nitrogen metabolism in cereals, as well as assisting in the selection of lines that show high nutrient utilisation efficiencies to deploy in future breeding strategies, such studies are limited in oats and are even restricted for the more commonly studied cereals such as wheat and barley.

## CRediT authorship contribution statement

**J. William Allwood:** Conceptualization, Methodology, Validation, Formal analysis, Investigation, Data curation, Visualization, Supervision. **Pilar Martinez-Martin:** Methodology, Investigation, Resources. **Yun Xu:** Methodology, Software, Formal analysis, Data curation, Visualization. **Alexander Cowan:** Methodology, Investigation, Resources. **Simon Pont:** Investigation, Resources. **Irene Griffiths:** Investigation, Resources. **Julie Sungurtas:** Investigation, Resources. **Sarah Clarke:** Conceptualization, Resources. **Royston Goodacre:** Methodology, Software, Supervision. **Athole Marshall:** Conceptualization, Supervision, Funding acquisition. **Derek Stewart:** Conceptualization, Supervision, Funding acquisition. **Catherine Howarth:** Conceptualization, Methodology, Formal analysis, Investigation, Supervision, Funding acquisition.

## Declaration of Competing Interest

The authors declare that they have no known competing financial interests or personal relationships that could have appeared to influence the work reported in this paper.

## Appendix A Supplementary data

Supplementary figure 1 Principal Components Analysis (PCA) scores plots (a) of GC-MS and LC-MS datasets for all three trials. PC Principal Component; ESI- LC-MS electrospray ionisation negative mode; ESI+ LC-MS electrospray ionisation positive mode.

Supplementary figure 2 Multi Block-Hierarchical Principal Components Analysis (MB-HPCA) of GC-MS and LC-MS datasets for the IBERS 2014 trial: (a) oat variety-based blocking (nitrogen trend) block scores plot; (b) oat variety-based blocking (nitrogen trend) super-scores and loadings plot; (c) nitrogen level-based blocking (variety trend) block scores plot; (d) nitrogen level-based blocking (variety trend) super-scores and loadings plot. Within the variety-based blocking MB-HPCA loadings plots (b), the red arrow indicates the cut off applied for metabolites that increase in concentration in response to nitrogen, the blue arrow indicates the cut off applied for metabolites that decrease in concentration in response to nitrogen. Within the nitrogen level-based blocking loadings plots (d), the blue arrow indicates the cut off applied for metabolites that are of higher levels in the dwarf variety, Balado, the red arrow indicates the cut off applied for metabolites that are of a higher level in the non-dwarf varieties. PC Principal Component. Nitrogen: level 1 basal; level 2 50 kg N/ha; level 3 100 kg N/ha; level 4 150 kg N/ha; level 5 200 kg N/ha.

Supplementary figure 3 Multi Block-Hierarchical Principal Components Analysis (MB-HPCA) of GC-MS and LC-MS datasets for the ADAS 2014 trial: (a) oat variety-based blocking (nitrogen trend) block scores plot; (b) oat variety-based blocking (nitrogen trend) super-scores and loadings plot; (c) nitrogen level-based blocking (variety trend) block scores plot; (d) nitrogen level-based blocking (variety trend) super-scores and loadings plot. Within the variety-based blocking loadings plots (b), the red arrow indicates the cut off applied for metabolites that increase in concentration in response to nitrogen, the blue arrow indicates the cut off applied for metabolites that decrease in concentration in response to nitrogen. Within the nitrogen level-based blocking loadings plots (d), the blue arrow indicates the cut off applied for metabolites that are of higher levels in the dwarf variety, Balado, the red arrow indicates the cut off applied for metabolites that are of a higher level in the non-dwarf varieties. PC Principal Component. Nitrogen: level 1 basal; level 2 50 kg N/ha; level 3 100 kg N/ha; level 4 150 kg N/ha; level 5 200 kg N/ha; level 6 250 kg N/ha.

Supplementary figure 4 Multi Block-Hierarchical Principal Components Analysis (MB-HPCA) of GC-MS and LC-MS datasets for the ADAS 2015 trial: (a) oat variety-based blocking (nitrogen trend) block scores plot; (b) oat variety-based blocking (nitrogen trend) super-scores and loadings plot; (c) nitrogen level-based blocking (variety trend) block scores plot; (d) nitrogen level-based blocking (variety trend) super-scores and loadings plot. Within the variety-based blocking loadings plots (b), the red arrow indicates the cut off applied for metabolites that increase in concentration in response to nitrogen, the blue arrow indicates the cut off applied for metabolites that decrease in concentration in response to nitrogen. Within the nitrogen level-based blocking loadings plots (d), the blue arrow indicates the cut off applied for metabolites that are of higher levels in the dwarf variety, Balado, the red arrow indicates the cut off applied for metabolites that are of a higher level in the non-dwarf varieties. PC Principal Component. Nitrogen: level 1 basal; level 2 60 kg N/ha; level 3 120 kg N/ha; level 4 180 kg N/ha; level 5 230 kg N/ha; level 6 280 kg N/ha.

Supplementary figure 5 Metabolite differences between oat variety. Heat map of log10 scaled normalised metabolite peak areas: (a) Central metabolites; (b) Lipids; (c) Secondary metabolites.

Supplementary table 1 Nitrogen field trial design. Location, year, oat varieties, basal nitrogen level, nitrogen applications and dates of application.

Supplementary table 2 Ultra High Performance Liquid Chromatography – Mass Spectrometry (ESI negative mode) compound retention time, analyte accurate mass (m/z), MS/MS fragmentation and identification. MultiBlock Hierarchical Principal Component Analysis (variety and nitrogen blocking statements) loadings (PC1 and PC2), nitrogen and variety based two-way ANOVA (Friedman test) p values following 5% False Discovery Rate (FDR) correction (Benjamini-Hochberg procedure), Spearmans Rho and associated p values.

Supplementary table 3 Ultra High Performance Liquid Chromatography – Mass Spectrometry (ESI positive mode) compound retention time, analyte accurate mass (m/z), MS/MS fragmentation and identification. MultiBlock Hierarchical Principal Component Analysis (variety and nitrogen blocking statements) loadings (PC1 and PC2), nitrogen and variety based two-way ANOVA (Friedman test) p values following 5% False Discovery Rate (FDR) correction (Benjamini-Hochberg procedure), Spearmans Rho and associated p values.

Supplementary table 4 Gas Chromatography – Mass Spectrometry compound retention index and identification. MultiBlock Hierarchical Principal Component Analysis (variety and nitrogen blocking statements) loadings (PC1 and PC2), nitrogen and variety based two-way ANOVA (Friedman test) p values following 5% False Discovery Rate (FDR) correction (Benjamini-Hochberg procedure), Spearmans Rho and associated p values.
