# Supplementary figures and images for: Assessing the impact of nitrogen supplementation in oats across multiple growth locations and years with targeted phenotyping and high-resolution metabolite profiling approaches
Source: Food Chem. 2021 Sep 1;355:129585. doi: 10.1016/j.foodchem.2021.129585 (PMC8121753; doi:10.1016/j.foodchem.2021.129585)

## Slide 1
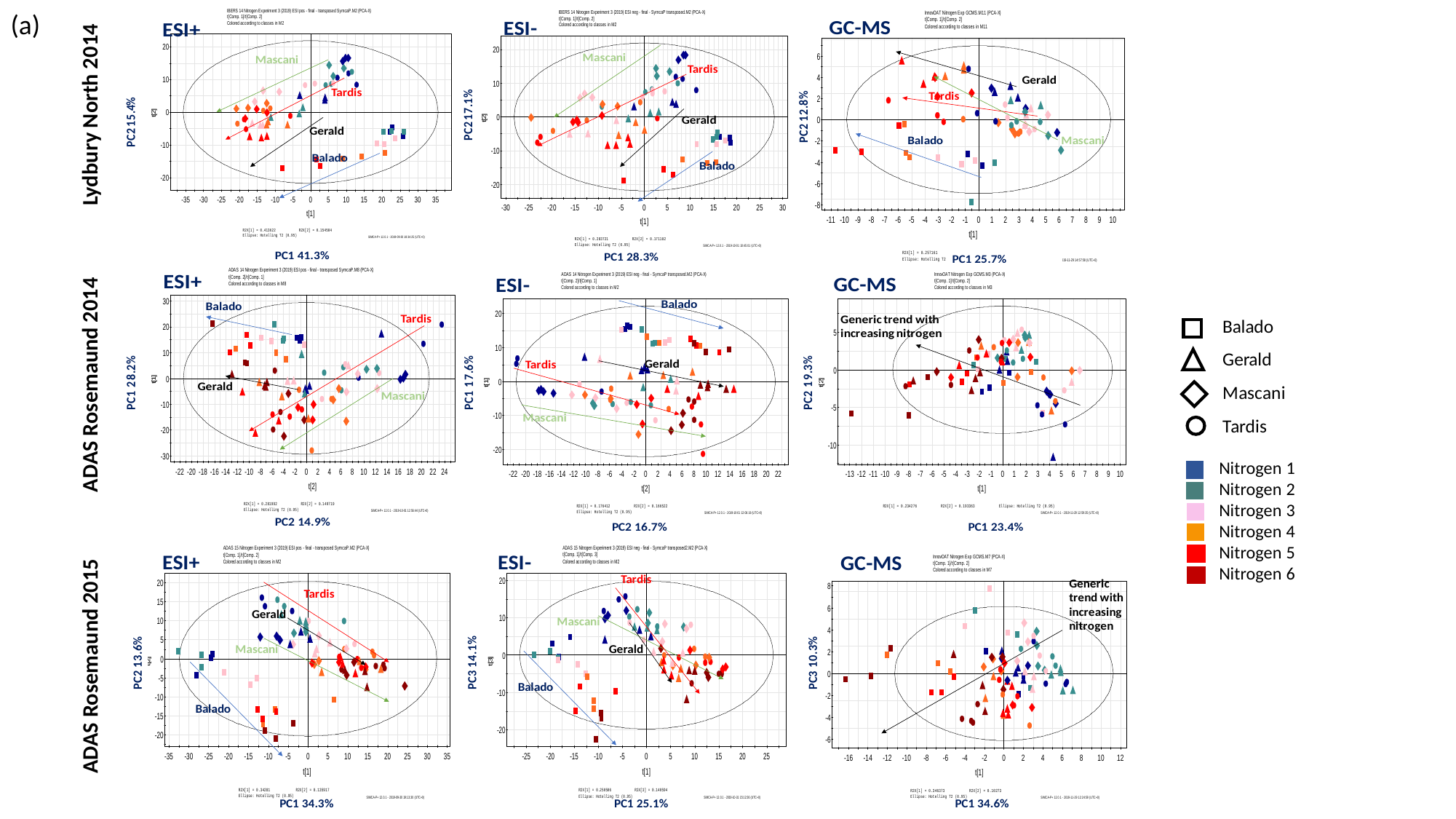

(a)

Supplement: Supplementary figure 1 — Principal Components Analysis (PCA) scores plots (a) of GC-MS and LC-MS datasets for all three trials. PC Principal Component; ESI- LC-MS electrospray ionisation negative mode; ESI+ LC-MS electrospray ionisation positive mode. [file mmc1.pptx]

## Slide 1
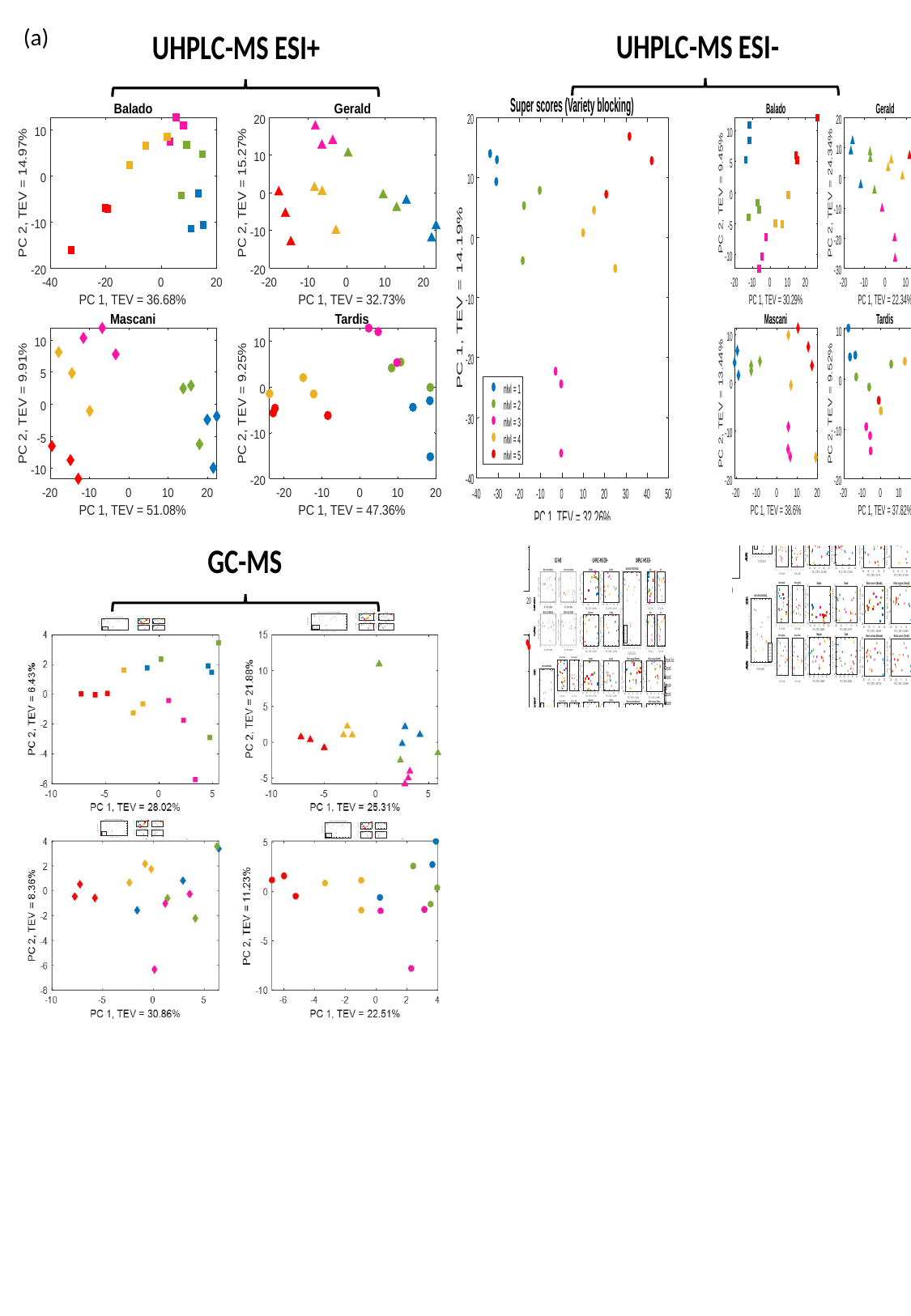

(a)

## Slide 2
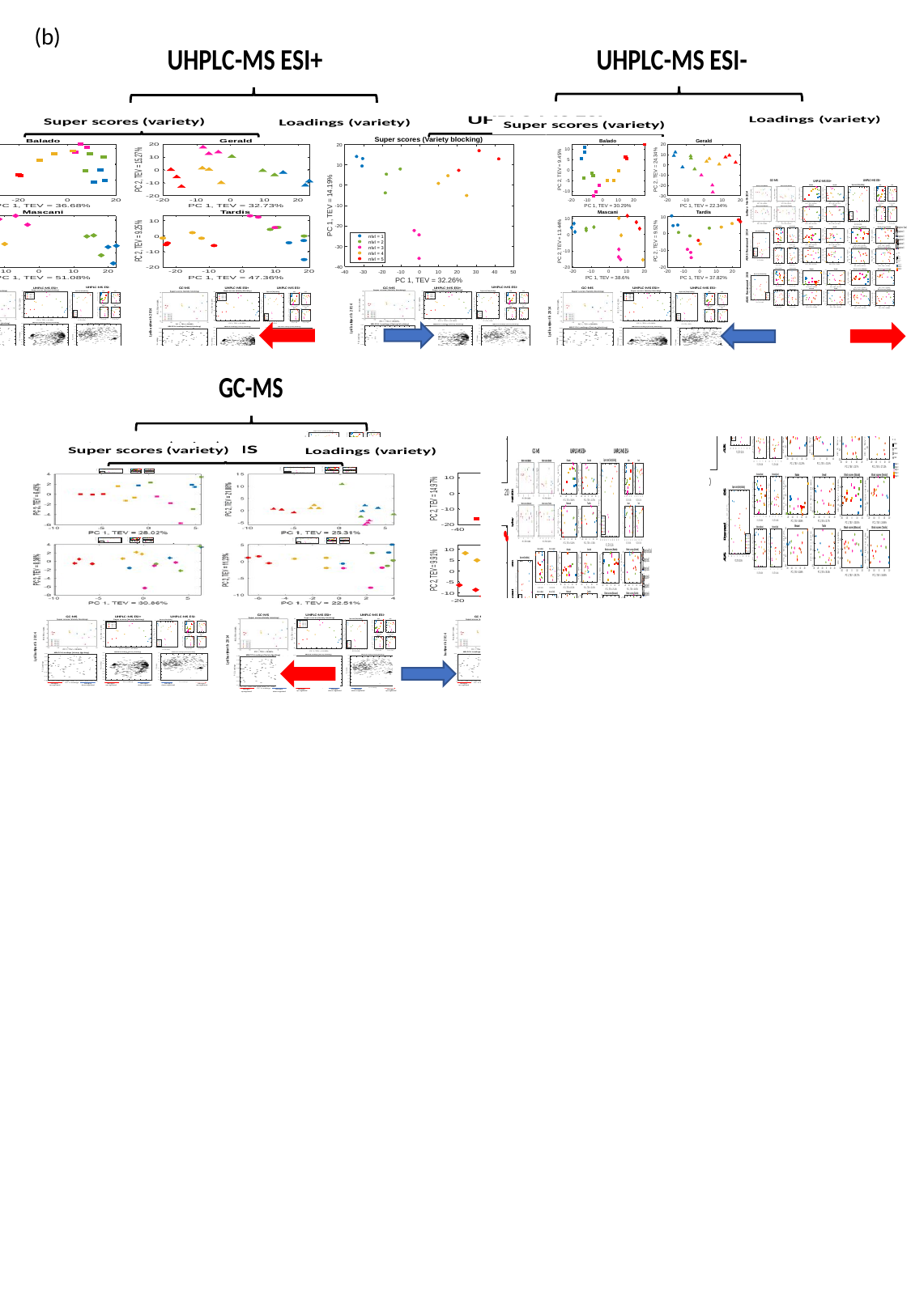

(b)

## Slide 3
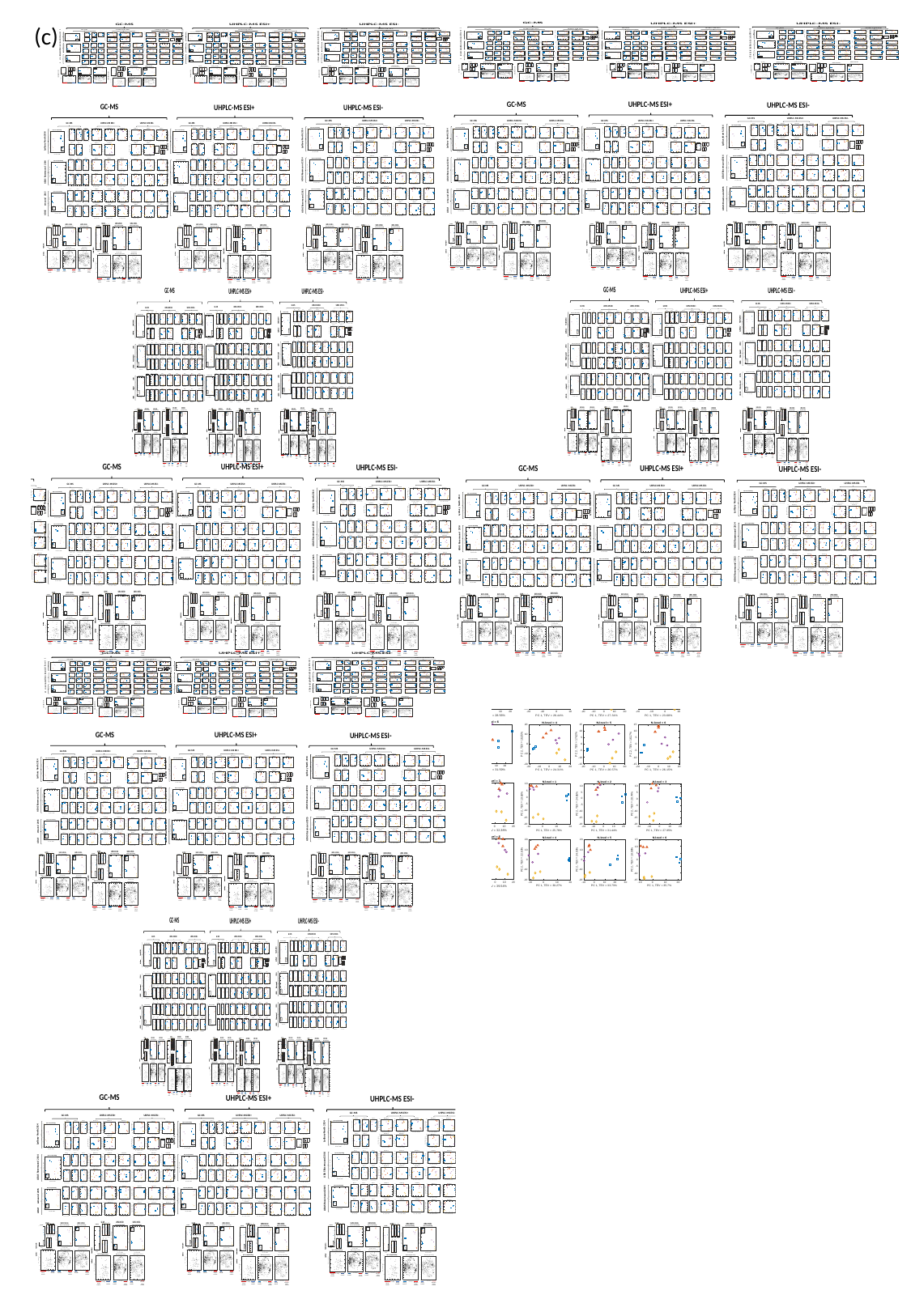

(c)

## Slide 4
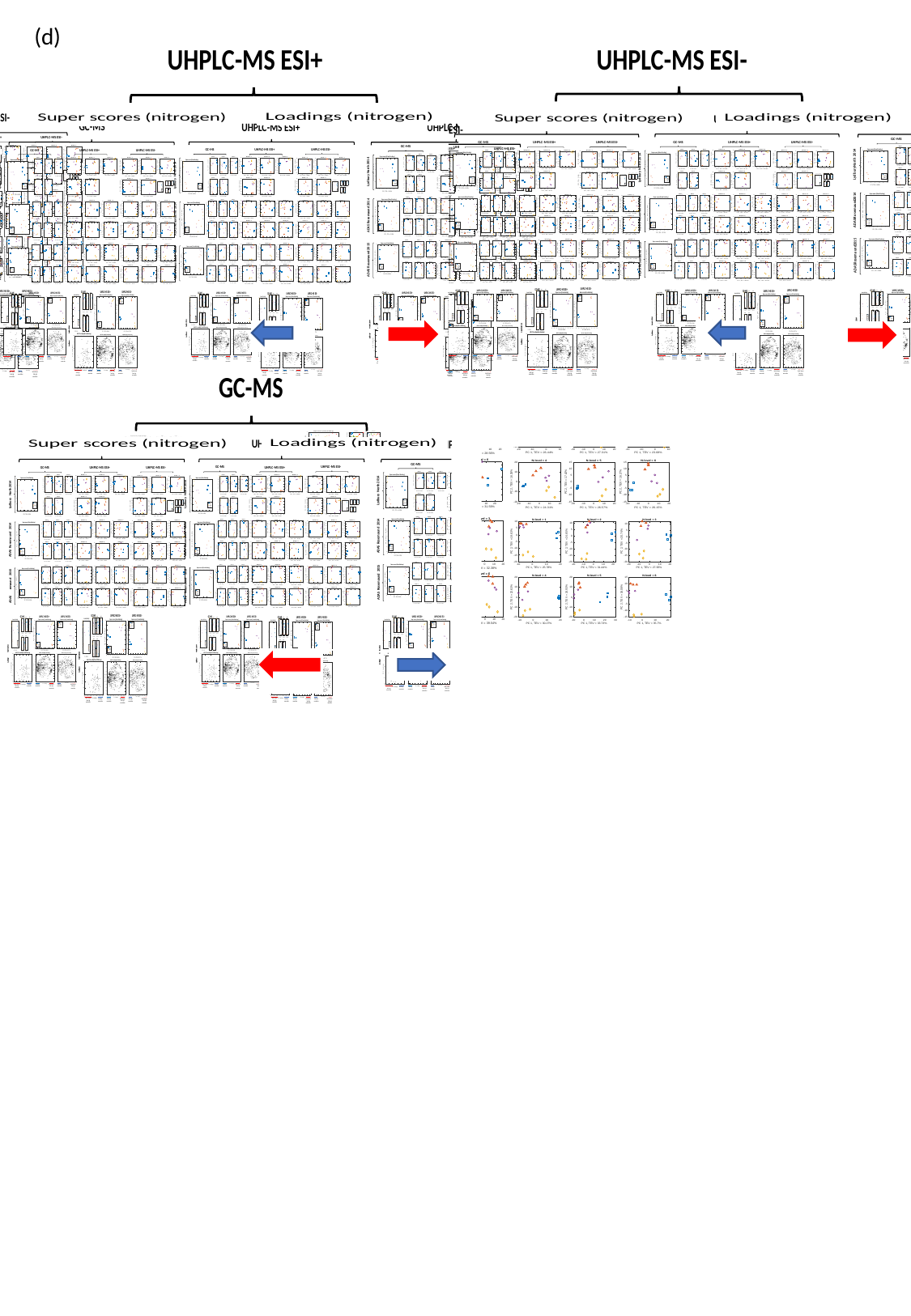

(d)

Supplement: Supplementary figure 2 — Multi Block-Hierarchical Principal Components Analysis (MB-HPCA) of GC-MS and LC-MS datasets for the IBERS 2014 trial: (a) oat variety-based blocking (nitrogen trend) block scores plot; (b) oat variety-based blocking (nitrogen trend) super-scores and loadings plot; (c) nitrogen level-based blocking (variety trend) block scores plot; (d) nitrogen level-based blocking (variety trend) super-scores and loadings plot. Within the variety-based blocking MB-HPCA loadings plots (b), the red arrow indicates the cut off applied for metabolites that increase in concentration in response to nitrogen, the blue arrow indicates the cut off applied for metabolites that decrease in concentration in response to nitrogen. Within the nitrogen level-based blocking loadings plots (d), the blue arrow indicates the cut off applied for metabolites that are of higher levels in the dwarf variety, Balado, the red arrow indicates the cut off applied for metabolites that are of a higher level in the non-dwarf varieties. PC Principal Component. Nitrogen: level 1 basal; level 2 50 kg N/ha; level 3 100 kg N/ha; level 4 150 kg N/ha; level 5 200 kg N/ha. [file mmc2.pptx]

## Slide 1
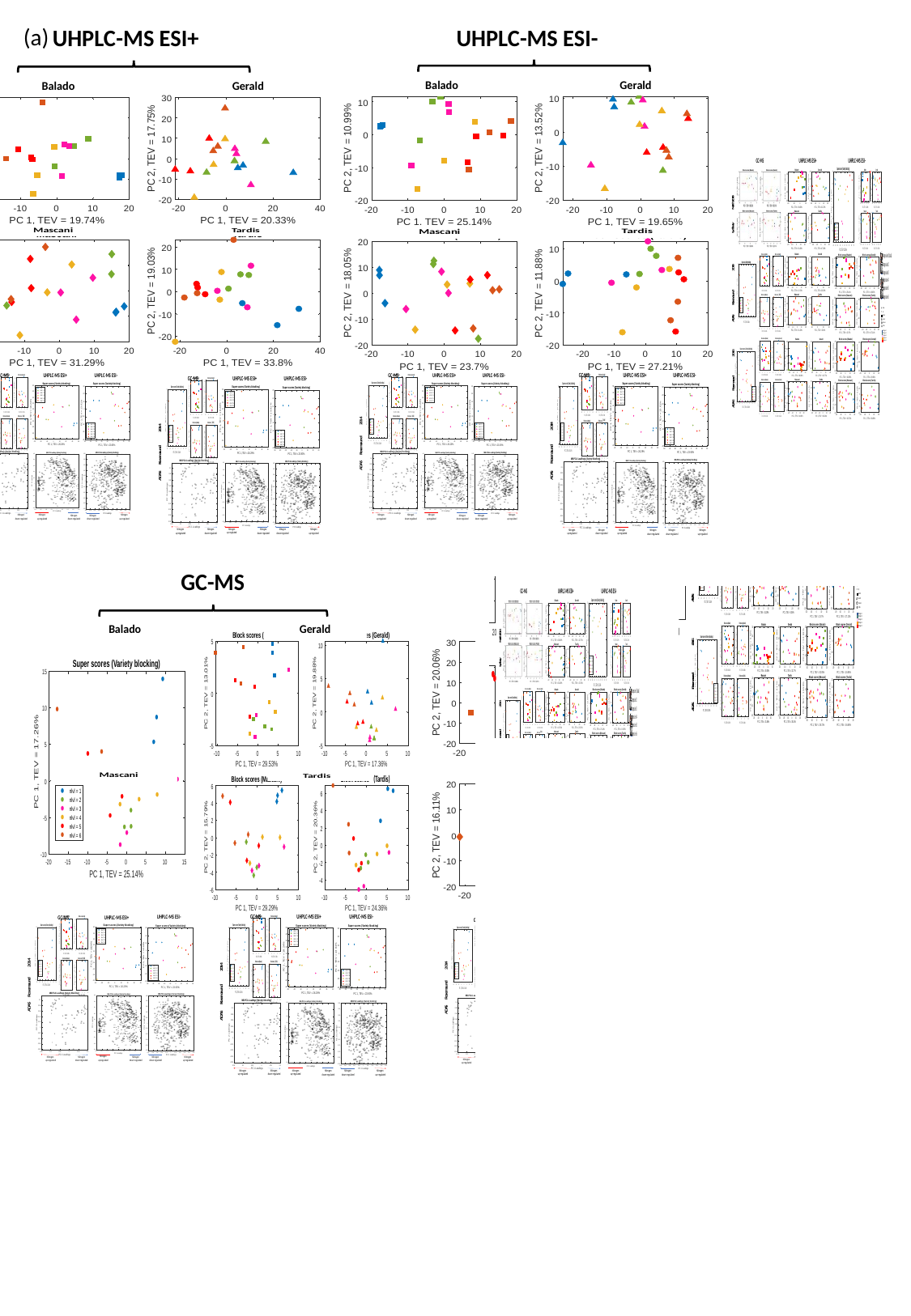

(a)

## Slide 2
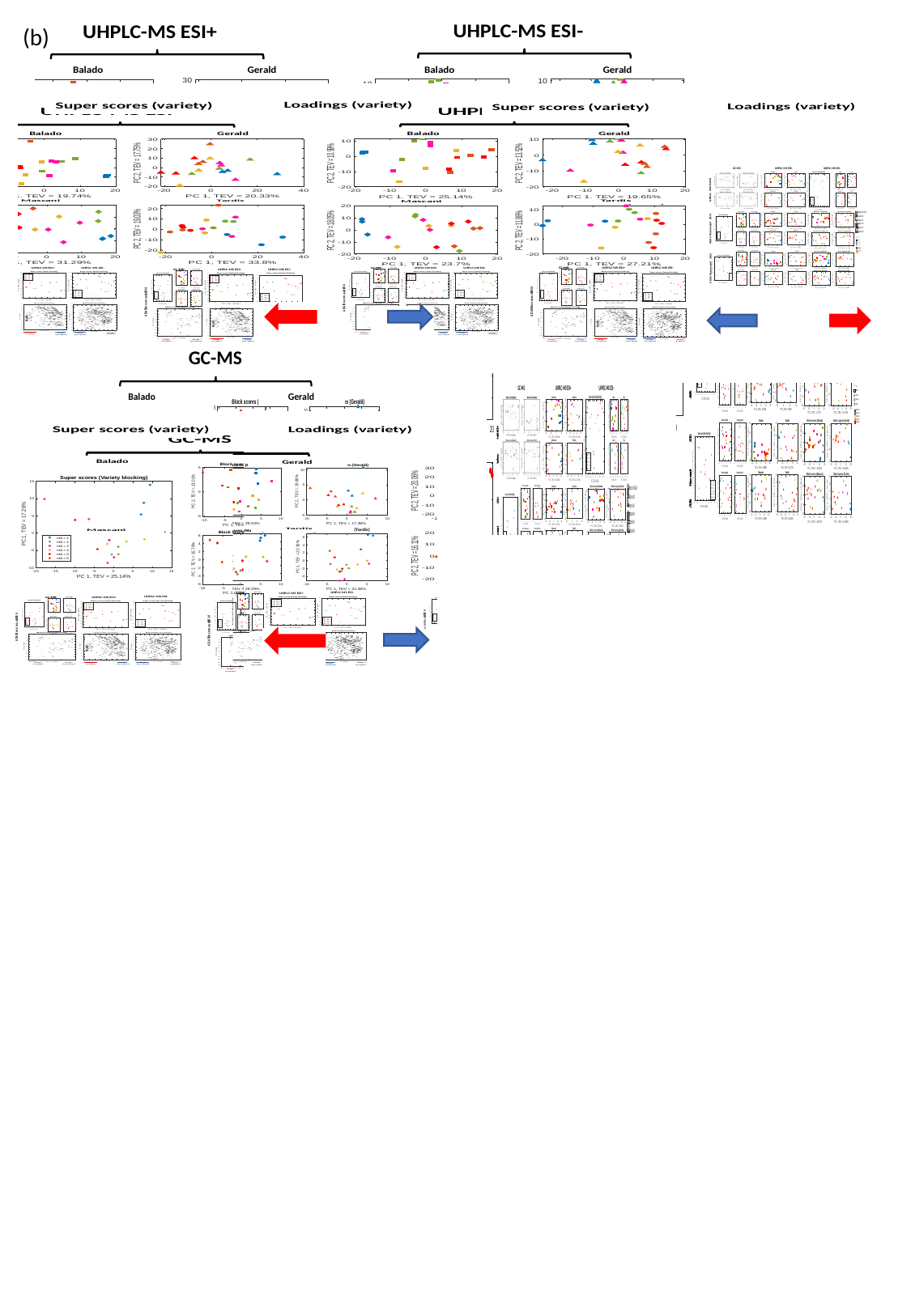

(b)

## Slide 3
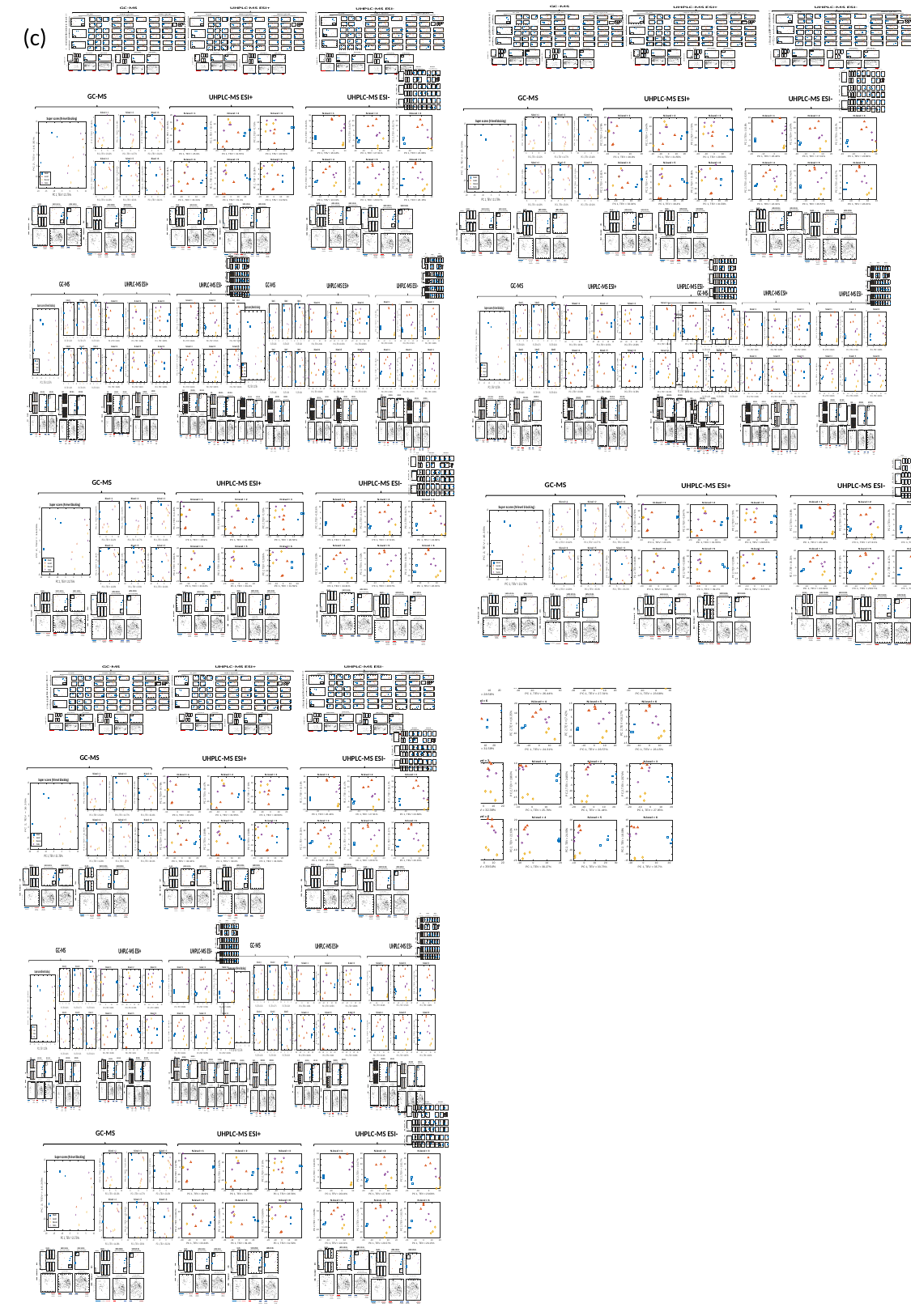

(c)

## Slide 4
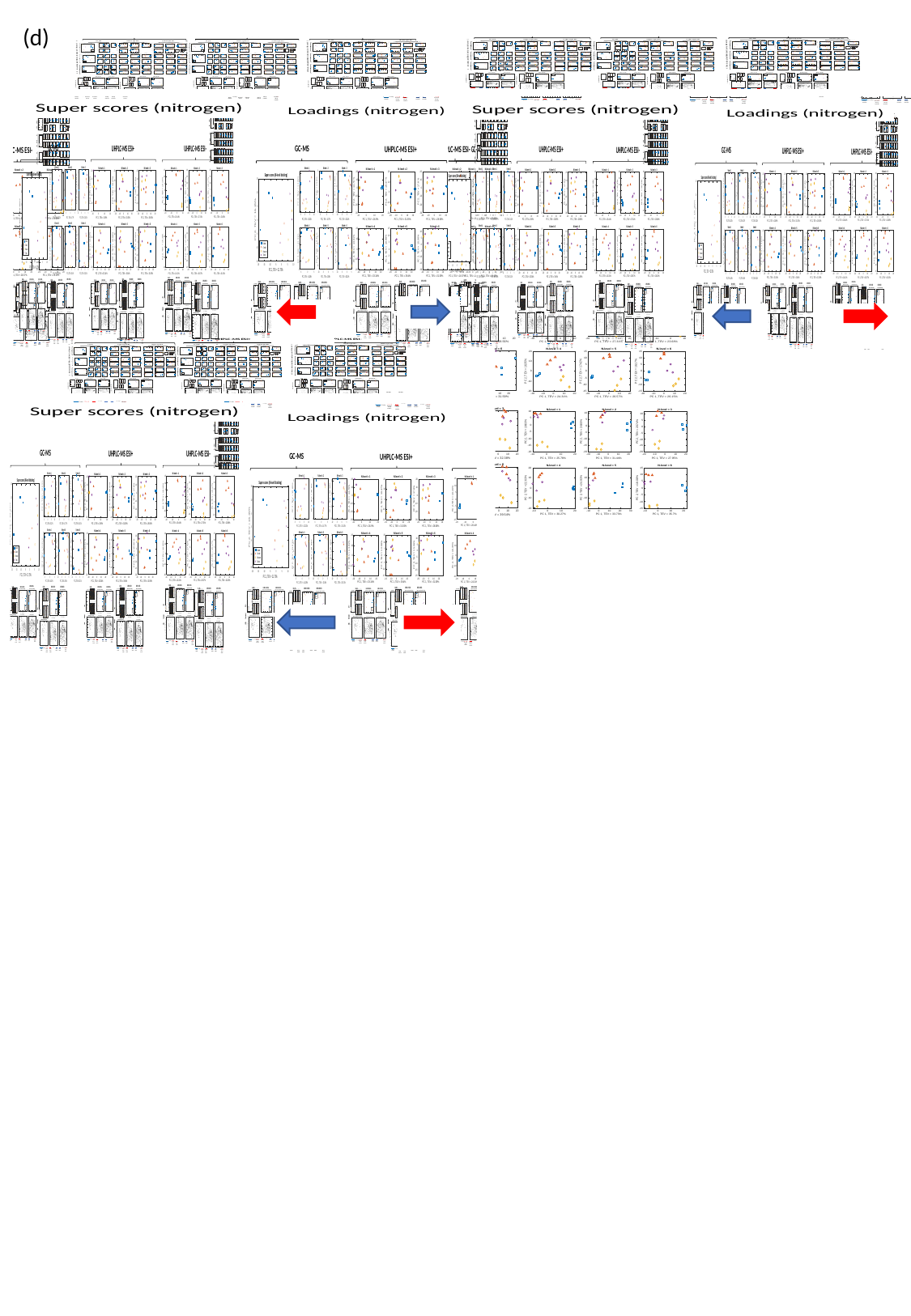

(d)

Supplement: Supplementary figure 3 — Multi Block-Hierarchical Principal Components Analysis (MB-HPCA) of GC-MS and LC-MS datasets for the ADAS 2014 trial: (a) oat variety-based blocking (nitrogen trend) block scores plot; (b) oat variety-based blocking (nitrogen trend) super-scores and loadings plot; (c) nitrogen level-based blocking (variety trend) block scores plot; (d) nitrogen level-based blocking (variety trend) super-scores and loadings plot. Within the variety-based blocking loadings plots (b), the red arrow indicates the cut off applied for metabolites that increase in concentration in response to nitrogen, the blue arrow indicates the cut off applied for metabolites that decrease in concentration in response to nitrogen. Within the nitrogen level-based blocking loadings plots (d), the blue arrow indicates the cut off applied for metabolites that are of higher levels in the dwarf variety, Balado, the red arrow indicates the cut off applied for metabolites that are of a higher level in the non-dwarf varieties. PC Principal Component. Nitrogen: level 1 basal; level 2 50 kg N/ha; level 3 100 kg N/ha; level 4 150 kg N/ha; level 5 200 kg N/ha; level 6 250 kg N/ha. [file mmc3.pptx]

## Slide 1
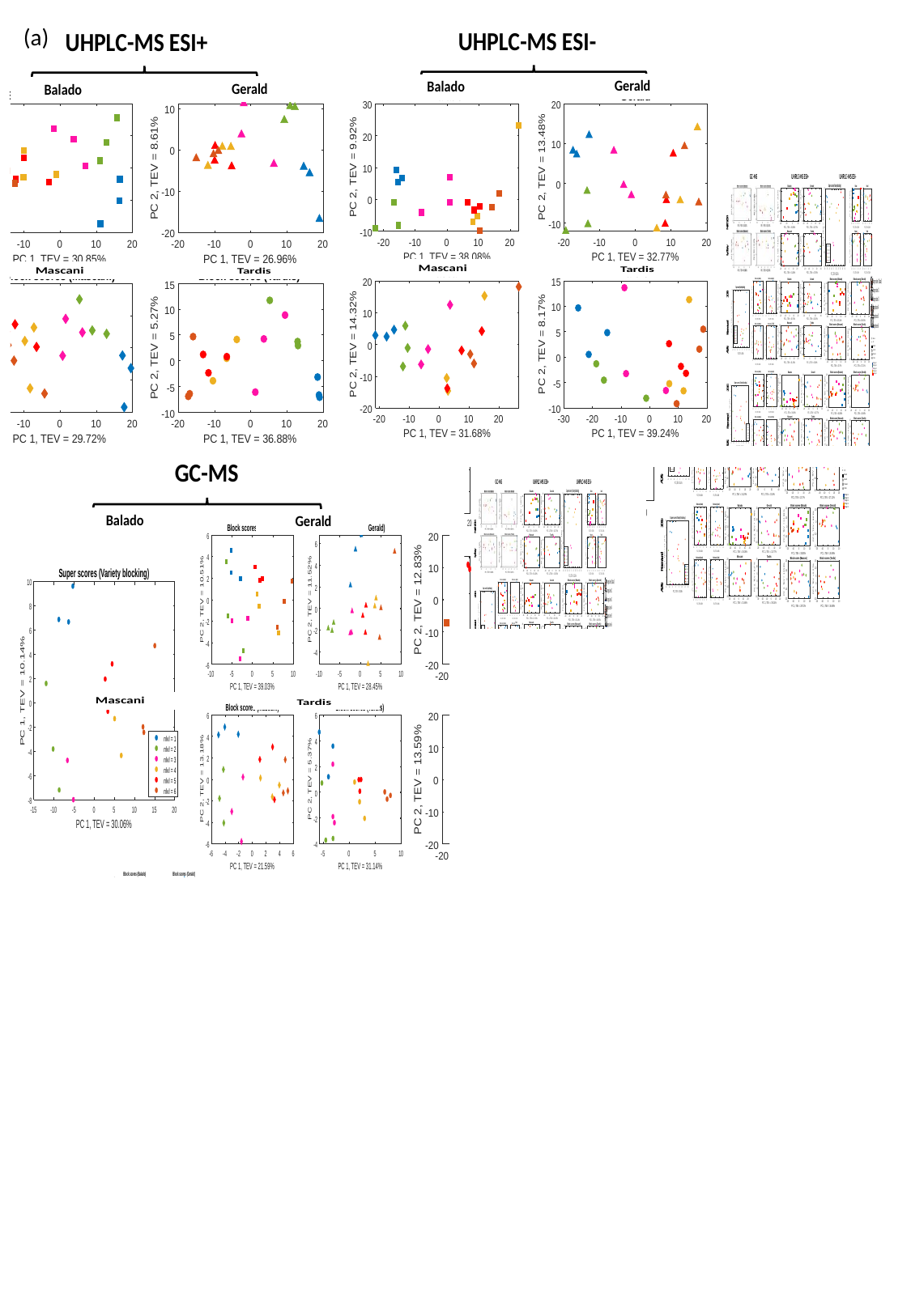

(a)

## Slide 2
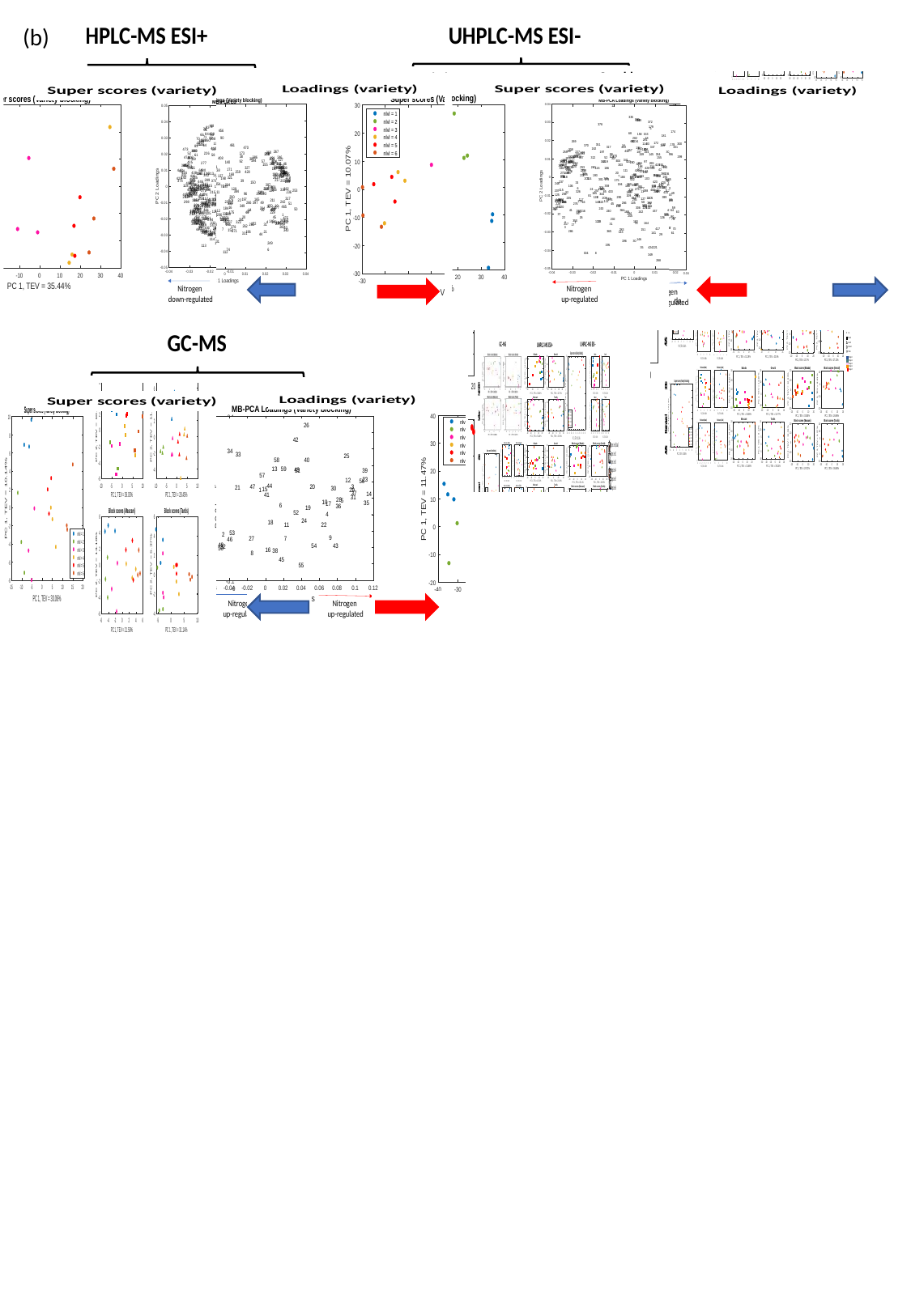

(b)

## Slide 3
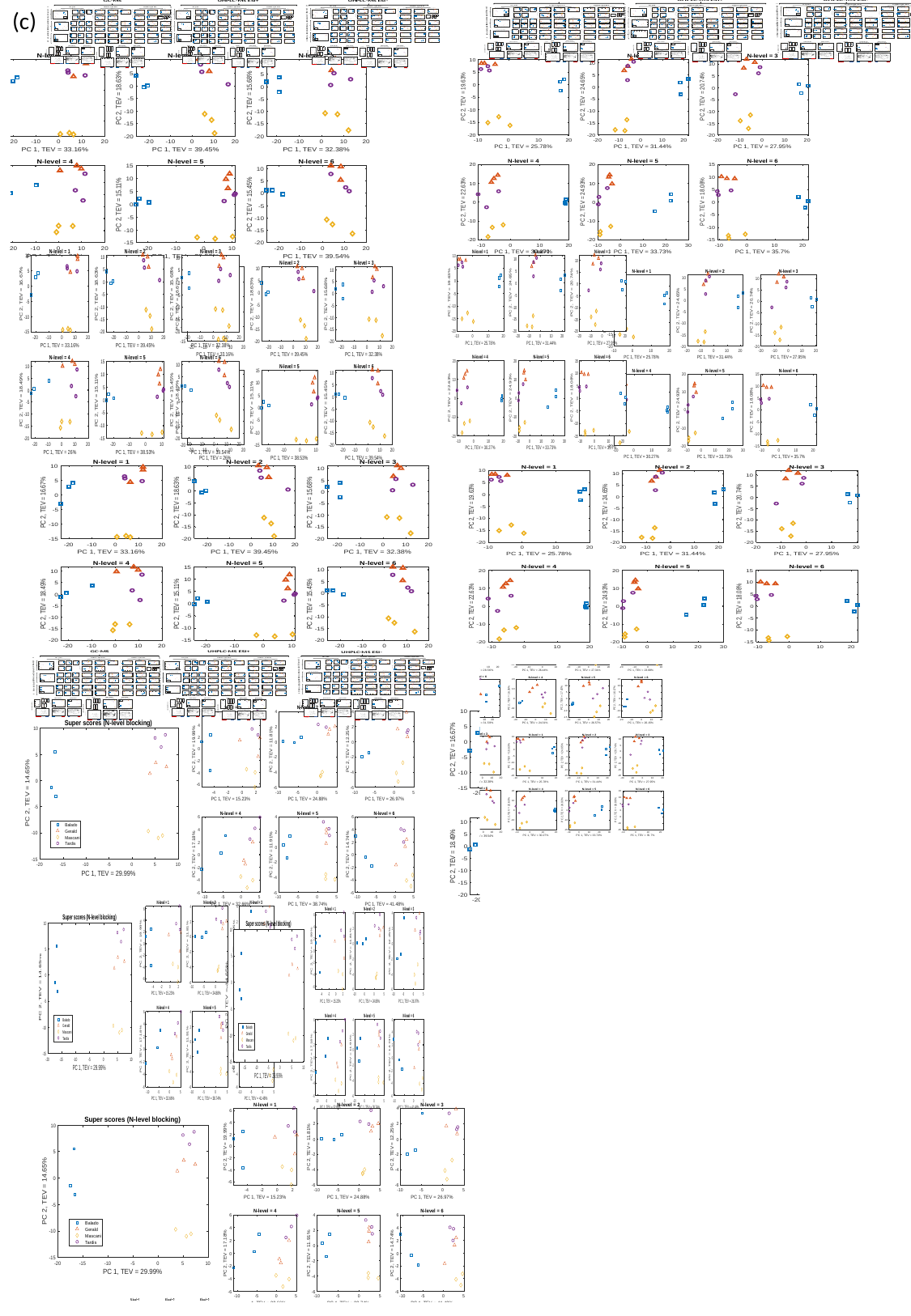

(c)

## Slide 4
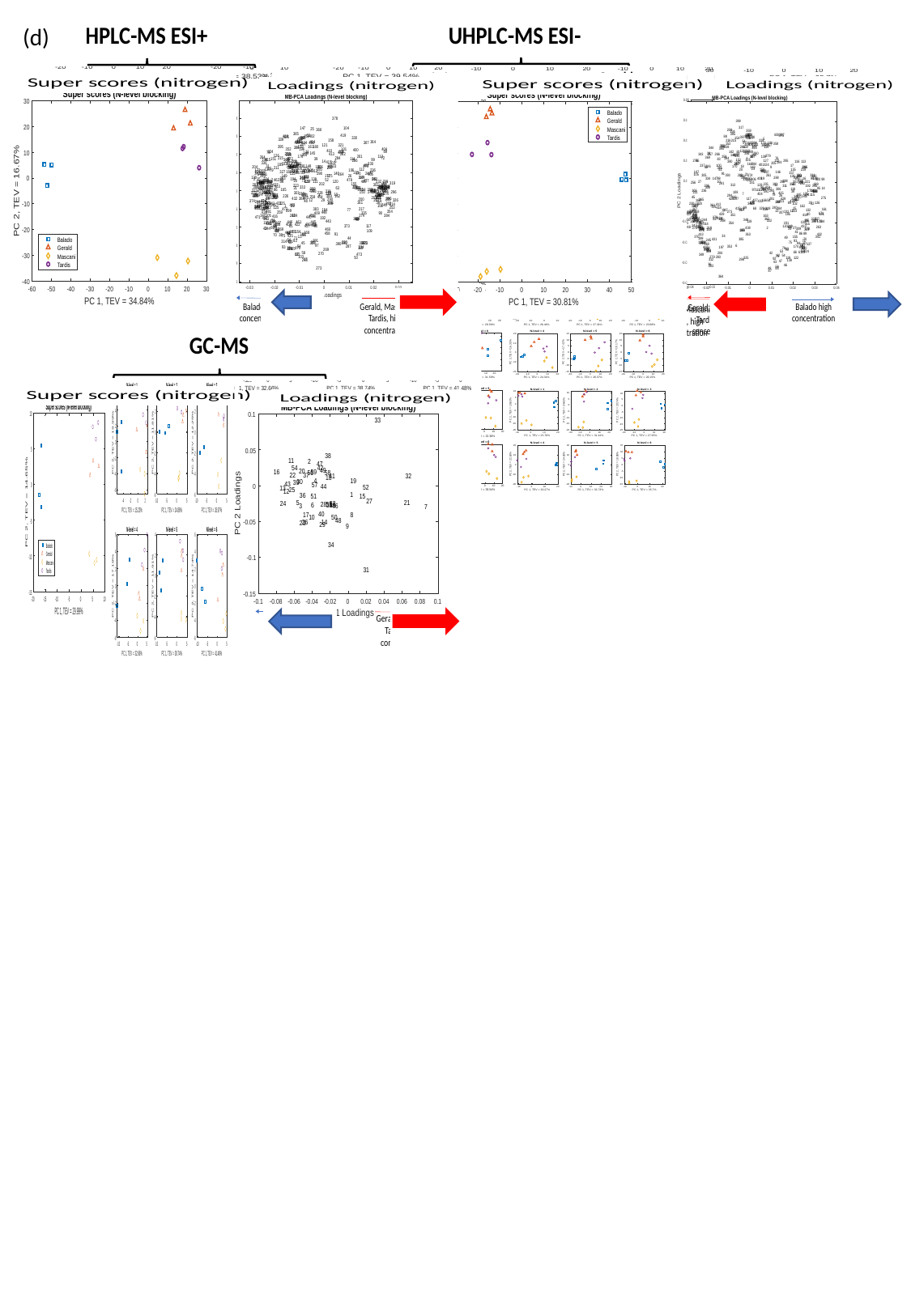

(d)

Supplement: Supplementary figure 4 — Multi Block-Hierarchical Principal Components Analysis (MB-HPCA) of GC-MS and LC-MS datasets for the ADAS 2015 trial: (a) oat variety-based blocking (nitrogen trend) block scores plot; (b) oat variety-based blocking (nitrogen trend) super-scores and loadings plot; (c) nitrogen level-based blocking (variety trend) block scores plot; (d) nitrogen level-based blocking (variety trend) super-scores and loadings plot. Within the variety-based blocking loadings plots (b), the red arrow indicates the cut off applied for metabolites that increase in concentration in response to nitrogen, the blue arrow indicates the cut off applied for metabolites that decrease in concentration in response to nitrogen. Within the nitrogen level-based blocking loadings plots (d), the blue arrow indicates the cut off applied for metabolites that are of higher levels in the dwarf variety, Balado, the red arrow indicates the cut off applied for metabolites that are of a higher level in the non-dwarf varieties. PC Principal Component. Nitrogen: level 1 basal; level 2 60 kg N/ha; level 3 120 kg N/ha; level 4 180 kg N/ha; level 5 230 kg N/ha; level 6 280 kg N/ha. [file mmc4.pptx]

## Slide 1
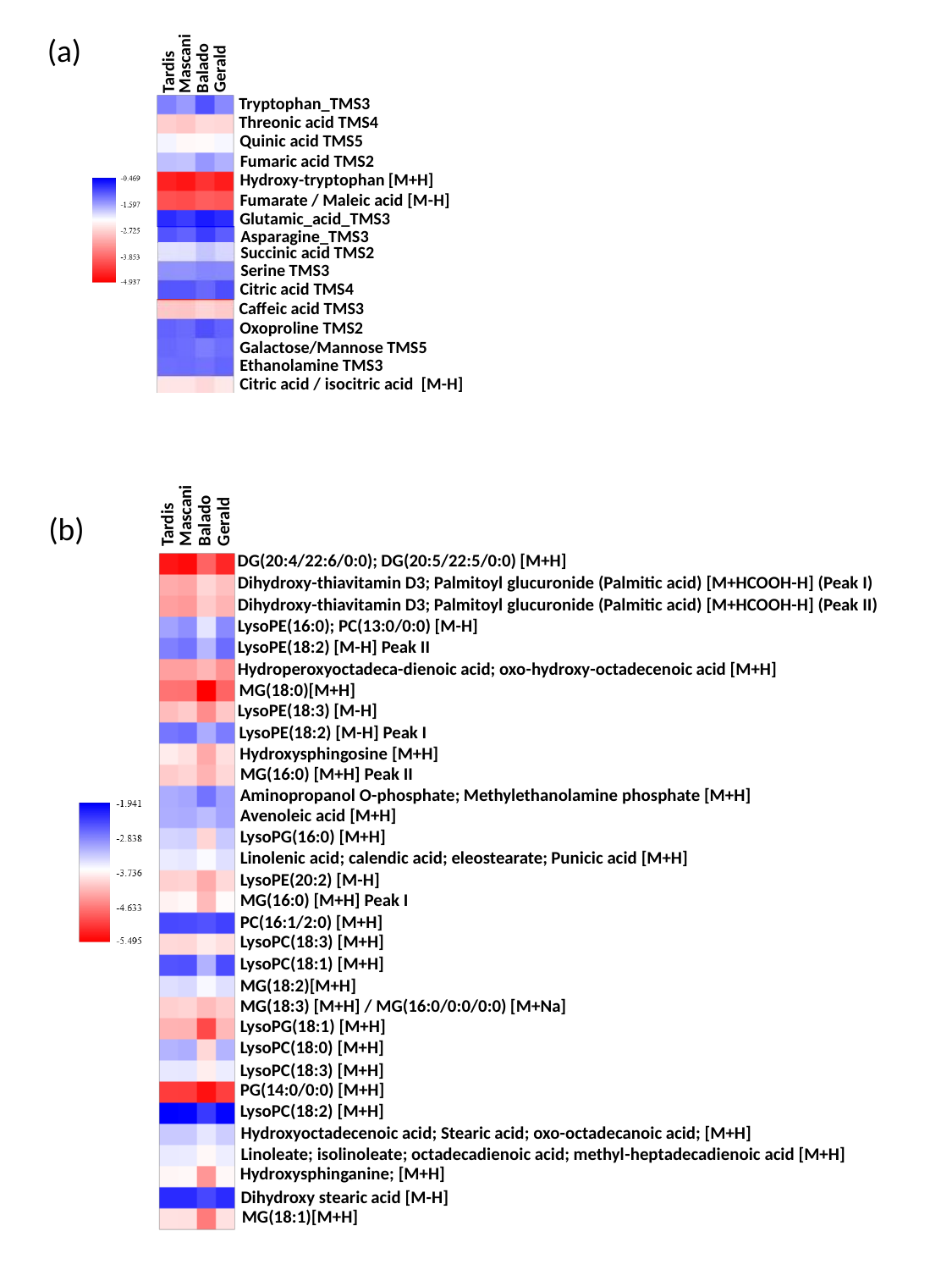

(a)
(b)

## Slide 2
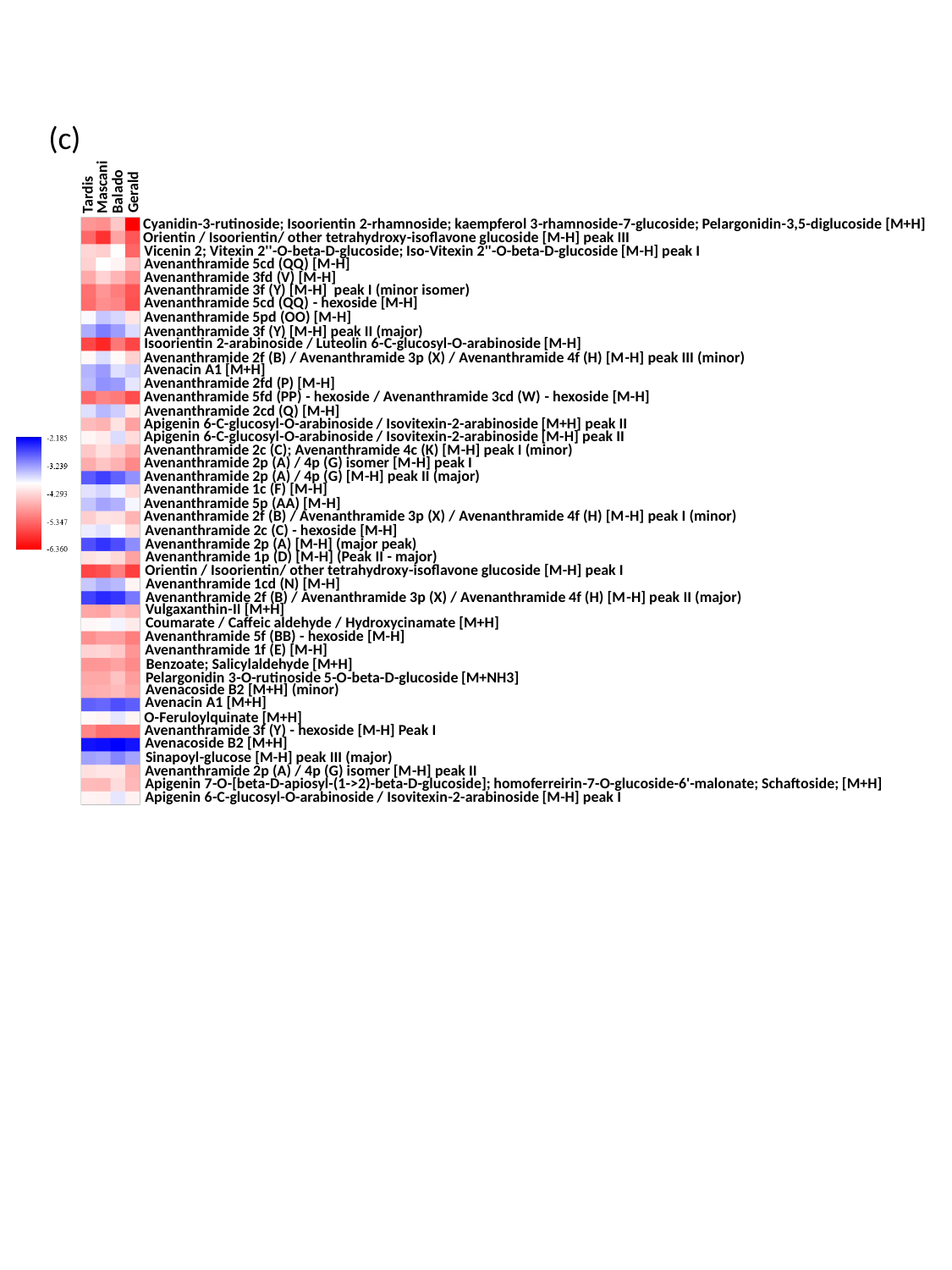

(c)

Supplement: Supplementary figure 5 — Metabolite differences between oat variety. Heat map of log10 scaled normalised metabolite peak areas: (a) Central metabolites; (b) Lipids; (c) Secondary metabolites. [file mmc5.pptx]
